# Post-mortem histopathology underlying β-amyloid PET imaging following flutemetamol F 18 injection

**DOI:** 10.1186/s40478-016-0399-z

**Published:** 2016-12-12

**Authors:** Milos D. Ikonomovic, Chris J. Buckley, Kerstin Heurling, Paul Sherwin, Paul A. Jones, Michelle Zanette, Chester A. Mathis, William E. Klunk, Aruna Chakrabarty, James Ironside, Azzam Ismail, Colin Smith, Dietmar R. Thal, Thomas G. Beach, Gill Farrar, Adrian P. L. Smith

**Affiliations:** 1 Department of Neurology, University of Pittsburgh, Pittsburgh, PA 15213 USA; 2 GE Healthcare, The Grove Centre (GC18), White Lion Road, Amersham, Buckinghamshire HP7 9LL UK; 3 GE Healthcare, 75184 Uppsala, Sweden; 4 Department of Surgical Sciences, Uppsala University, 75185 Uppsala, Sweden; 5 GE Healthcare, Marlborough, MA 01752 USA; 6 Department of Radiology, University of Pittsburgh, Pittsburgh, PA 15213 USA; 7 Department of Psychiatry, University of Pittsburgh, Pittsburgh, PA 15213 USA; 8 Pathology and Tumour Biology, Leeds Institute of Molecular Medicine, St James’s University Hospital, Leeds, UK; 9 National CJD Research & Surveillance Unit University of Edinburgh Western General Hospital Edinburgh, EH4 2XU Edinburgh, UK; 10 Academic Department of Neuropathology, Centre for Clinical Brain Sciences, Edinburgh, EH16 4SB UK; 11 Department of Neuroscience - Laboratory for Neuropathology, KU-Leuven, Leuven, Belgium; 12 Banner Sun Health Research Institute, Sun City, AZ 85351 USA; 13 Geriatric Research Education and Clinical Center, VA Pittsburgh Healthcare System, Pittsburgh, USA; 14 Department of Pathology, UZ Leuven, Leuven, Belgium

**Keywords:** Flutemetamol, PET, Amyloid, Alzheimer’s disease, Neuropathology (4-6 allowed)

## Abstract

In vivo imaging of fibrillar β-amyloid deposits may assist clinical diagnosis of Alzheimer’s disease (AD), aid treatment selection for patients, assist clinical trials of therapeutic drugs through subject selection, and be used as an outcome measure. A recent phase III trial of [^18^F]flutemetamol positron emission tomography (PET) imaging in 106 end-of-life subjects demonstrated the ability to identify fibrillar β-amyloid by comparing in vivo PET to post-mortem histopathology. Post-mortem analyses demonstrated a broad and continuous spectrum of β-amyloid pathology in AD and other dementing and non-dementing disease groups. The GE067-026 trial demonstrated 91% sensitivity and 90% specificity of [^18^F]flutemetamol PET by majority read for the presence of moderate or frequent plaques. The probability of an abnormal [^18^F]flutemetamol scan increased with neocortical plaque density and AD diagnosis. All dementia cases with non-AD neurodegenerative diseases and those without histopathological features of β-amyloid deposits were [^18^F]flutemetamol negative. Majority PET assessments accurately reflected the amyloid plaque burden in 90% of cases. However, ten cases demonstrated a mismatch between PET image interpretations and post-mortem findings. Although tracer retention was best associated with amyloid in neuritic plaques, amyloid in diffuse plaques and cerebral amyloid angiopathy best explain three [^18^F]flutemetamol positive cases with mismatched (sparse) neuritic plaque burden. Advanced cortical atrophy was associated with the seven false negative [^18^F]flutemetamol images. The interpretation of images from pathologically equivocal cases was associated with low reader confidence and inter-reader agreement. Our results support that amyloid in neuritic plaque burden is the primary form of β-amyloid pathology detectable with [^18^F]flutemetamol PET imaging. ClinicalTrials.gov NCT01165554. Registered June 21, 2010; NCT02090855. Registered March 11, 2014.

**Electronic supplementary material:**

The online version of this article (doi:10.1186/s40478-016-0399-z) contains supplementary material, which is available to authorized users.

## Introduction

The relatively modest accuracy of a clinical diagnosis of Alzheimer’s disease (AD) when compared to the definitive neuropathological findings at autopsy [4] demonstrates a current unmet need to detect the neuropathological hallmarks of AD (β-amyloid plaques and neurofibrillary tangles) in life. Plaques containing fibrillar β-amyloid are readily detectable in histological tissue specimens using dyes with high affinity for amyloid β-sheet structure (Thioflavin-S and Congo red). Analogues of these dyes have been radiolabelled to create positron emission tomography (PET) imaging tracers for detecting β-amyloid plaques in vivo. PET amyloid tracers such as Pittsburgh compound B ([^11^C]PiB) and its derivative [^18^F]flutemetamol (Vizamyl^TM^) generally can distinguish between the presence of moderate or frequent amyloid plaques, required for the diagnosis of AD, or lesser densities that would rule out AD [24, 61].

Results of recent studies using PET β-amyloid tracers have shown a good association between tracer retention and underlying β-amyloid plaques in brain autopsy and biopsy samples [11, 12, 28, 31, 35–38, 47, 50–52, 65–67]. However, many of these studies have drawn on a pool of end-of-life subjects with advanced disease, in which autopsy is anticipated shortly after PET imaging and with the specific goal to demonstrate efficacy. Furthermore these studies have been required to be measured against semiquantitative neuritic plaque counts as the historical standard of truth of amyloid burden. The criteria for subject selection in such studies is critical; selection of advanced dementia subjects and a group of subjects with preserved cognition will tend to give subject sets that are at the extreme ends of the β-amyloid spectrum (neuritic plaque densities of *none* or *frequent* by CERAD criteria [43]), biasing towards finding high test sensitivity and specificity. On the other hand, the likely beneficiaries of β-amyloid PET imaging would be those in early disease development with borderline β-amyloid pathology (*sparse* or *moderate* plaque densities by CERAD criteria). Such subjects may be most appropriate for trials testing disease-modifying drugs because they are more likely to respond to available treatments. Subjects with a limited life expectancy unrelated to cognitive status (e.g., elderly oncology patients) may have intermediate AD pathology, as well as preserved cognition.

In the recently reported [^18^F]flutemetamol multicentre end-of-life β-amyloid imaging trials [14, 49], subjects were drawn from both dementia clinics and hospice centres. While visual PET image assessments were compared to neuritic plaque burden to demonstrate efficacy for regulatory approval, neuropathology diagnoses and multiregional histometric measures were also recorded including amyloid plaque assessment by immunohistochemistry and amyloid phase analysis. Rather than a dichotomous distribution consisting mainly of pathology extremes, the 106 subjects in this study represent a broad and continuous spectrum of β-amyloid pathology, as well as coincident neuropathology. This enabled assessment of subjects with cortical β-amyloid burdens that were borderline for clinicopathological significance, associated with diffuse or neuritic plaques as well as the effect of mixed pathology which is the subject of the present study.

## Materials and methods

### Ethics, consent and permissions and consent to publish

This was a phase 3 multicenter PET study of flutemetamol injection labelled with radioactive fluorine 18 ([^18^F]flutemetamol) for detecting brain Aβ.

Institutional review boards or ethics committees at the following institutions approved the study protocol before initiation: St Margaret’s Hospital and Moorgreen Hospital (Hammersmith, Queen Charlotte's & Chelsea Research Ethics Committee); University of Michigan (The University of Michigan Medical School Institutional Review Board); Banner Alzheimer’s Institute, Banner Sun Health Research Institute, Premier Research Institute, Miami Jewish Health Systems, Galiz Research, VERITAS Research, LasVegas Radiology, Memory Enhancement Center and Compass Research (Western Institutional Review Board); Mt Sinai Medical Center, Wien Center for Alzheimer’s Disease, (Mount Sinai Medical Center); Michigan State University (Michigan State University, Biomedical and Health Institutional Review Board); Barrows Neurological Institute (St. Joseph's Hospital & Medical Center); Warren Alpert Medical School of Brown University (Lifespan Research Protection Office, RI, USA).

All participants or their legal representatives provided prior written informed consent including agreement to publish subject to anonymization.

### Trial subjects

Subjects with cognitive status ranging from normal to advanced dementia and with a life expectancy of 12 months or less were enrolled at 16 centres in the United States and the United Kingdom. Of 180 enrolled subjects who were given [^18^F]flutemetamol, 69 died within 13 months of imaging (Study GE067-007 [14]). Sixty-eight brains were evaluable for a) neuritic plaque assessment, b) correlation analysis between β-amyloid immunohistochemistry (IHC) and [^18^F]flutemetamol standardised uptake value ratio (SUVR) values, and c) neuropathology diagnoses. Additional deaths occurring after this time period but prior to July 2013 allowed a further 39 evaluable brains to be included in a re-read trial (Study GE067-026). One subject in GE067-007, who died shortly after an accidental fall, underwent a Medical Examiner’s autopsy, and this precluded regional mapping for the primary objectives. One GE067-026 brain was excluded as it was frozen rather than fixed at the site neuropathology laboratory. A total of 106 subjects were analysed in this study.

### Procedures

#### Tissue processing and sampling

The whole brain was removed at autopsy, with brain stem and cerebellum attached, and the two cerebral hemispheres were separated. The entire left hemisphere was immersion fixed in 10% neutral buffered formalin for 21 days prior to transport. Dissection, sampling, macroscopic photography and examination, histological processing and staining were performed at a single central laboratory (Covance Laboratories Ltd, Harrogate UK) to minimize processing variability. Upon receipt of the fixed hemisphere, the brain stem and hindbrain were removed by transverse section of the midbrain at the level of the third cranial nerve, and the hemisphere was cut into 1-cm coronal slices. Eight neocortical regions were sampled; the middle frontal gyrus, superior temporal gyrus, middle temporal gyrus, and inferior parietal lobe from the lateral cerebral surfaces (these regions are required by the Consortium to Establish a Registry for Alzheimer's Disease [CERAD] [43]) and the anterior cingulate gyrus, posterior cingulate gyri, precuneus, and primary visual cortex from the medial surface. Two 3-mm thick tissue blocks from the anterior and posterior surface of each 1-cm thick cortical sample were embedded in paraffin. Eleven additional paraffin tissue blocks were prepared to enable neuropathology diagnostics; i) olfactory tract and bulb, ii) striatum, iii) cholinergic basal forebrain nucleus, iv) thalamus, v) medulla, vi) pre- and post-central gyri, vii) anterior hippocampus, amygdala and entorhinal cortex, viii) hippocampal body, ix) pons, x) midbrain, and xi) cerebellum. The neuropathology diagnostic slide set consisted of sections from the eight cortical regions and the eleven additional regions; nineteen regions in total. Histological analysis of the neocortical regions was restricted to the left hemisphere, as the right hemisphere was sometimes retained frozen for biochemical analysis and was not always available. Prior evidence indicated that β-amyloid pathology is typically symmetric in AD [44]. Three sections each separated by approximately 100 μm were taken from both the anterior and posterior tissue blocks from each neocortical region.

### Neuritic plaque assessment

The scoring of neuritic plaque density has been a standard part of AD neuropathology since 1991 Mirra et al. [33] and through two iterations of recommended guidelines Hyman et al. [26], Hyman et al. [25] and were therefore deemed the most appropriate measure as the standard of truth to demonstrate efficacy for market authorisation regulatory approval when the GE067-007 Clinical trial was initiated and for other amyloid PET tracers [11, 12, 14]. Indeed, the regulatory authorities required the neuritic plaques assessment as described by Mirra et al. [43]. However, it is recognised that neuritic plaques are identified by dystrophic neurites (Tauopathy) to which an amyloid tracer is not expected to bind and that amyloid PET tracers bind to the amyloid component in the neuritic plaques.

Bielschowsky silver staining [39] was pre-validated from standard techniques as the primary *a priori* histopathology standard of truth (SOT) for the presence of clinically significant cortical β-amyloid. The potential for inter-subject variation in staining was mitigated by centralising the staining at a single laboratory, including appropriate quality controls and recording staining quality on case report forms. For all subjects, Bielschowsky-stained slides were assessed semiquantitatively for neuritic plaque frequency in neocortical regions using published CERAD-based protocols; this methodology supports the subject-level rating for neuritic plaque density determined by the highest density (none, sparse, moderate or frequent) found in any 10x objective field and in any neocortical region examined [43]. While the use of this SOT may be suitable for supporting a diagnosis of AD, other SOTs may be more suited for comparison to a more quantitative PET study. Accordingly, the classification of each case as normal or abnormal was determined by two additional methods (see Table 1), using multiple measures of neuritic plaque density in multiple regions. Multiple assessments mitigated focal heterogeneity that would be evident microscopically but not macroscopically at the lower (6-mm) resolution of PET imaging. The 2 multi-measure methods were both modified from CERAD and differed mainly in the number of regions assessed. The first of these modified assessments averaged numeric assessments (arithmetic mean - see below) of neuritic plaque density in each of the 8 neocortical regions to give a continuous variable. This method was aligned to the global cortical PET assessment regions and formed our Standard of Truth (mCERAD_SOT_). The second method limited the assessment to only those regions originally recommended by CERAD and retained the categorical assignation of ‘none’, ‘sparse’, ‘moderate’, or ‘frequent’ by employing the mode to average multiple categorical scores within regions (mCERAD_mode_), thus achieving a score more representative of the total area instead of being biased towards focal accumulations of plaques.

**Table 1:** Bielschowsky assessments of neuritic plaque densities

Algorithm	mCERAD_SOT_	mCERAD_mode_	CERAD
Regions assessed	MFL, MTG, STG, IPL, ACG, PCG, PRC, PVC	MFL, MTG, STG, IPL	MFL, MTG, STG, IPL
Regional assessments	8 regional averages (mean) of 30 intra-regional numeric^a^ assessments (each 0 ≤ x ≤ 3)	4 regional averages (mode) of 30 intra-regional categorical assessments (none, sparse, moderate, frequent)	1 global assessment from region of highest involvement (none, sparse, moderate, frequent)
Case dichotomy	Any region >1.5 is *abnormal*	Any region = moderate or frequent is *abnormal*	moderate or frequent is *abnormal*

*MFL* midfrontal lobe, *MTG* middle temporal gyrus, *STG* superior temporal gyrus, *IPL* inferior parietal lobe, *ACG* anterior cingulate gyrus, *PCG* posterior cingulate gyrus, *PRC* Precuneus, *PVC* primary visual cortex

^a^Each assessment was recorded as 0 = none (no plaques per 100x field of view), 1 = sparse (1-5 plaques), 2 = moderate (6-19 plaques) or 3 = frequent (20 or more plaques). A total of 240 cortical assessments were recorded for each case

For the two modified CERAD assessments, a total of 30 measures per cortical region were recorded on case report forms by neuropathologists (JI and CS). Briefly, 5 randomly selected grey-matter fields of view per section were assessed and neuritic plaque densities were recorded as a score of 0 = *none* (no plaques), 1 = *sparse* (1–5 plaques), 2 = *moderate* (6–19 plaques), and 3 = *frequent* (20+ plaques per 100x field of view [FoV]). [43, 60] For mCERAD_SOT_, 8 regional scores were calculated as the arithmetic mean of the 30 measures per region. The scale midpoint was 1.5, representing the threshold between sparse and moderate categories; a mean score ≤1.5 was considered normal while a mean score of >1.5 was considered abnormal for each region. The dichotomous classification of the whole brain as β-amyloid normal or abnormal was aligned to the CERAD principle of the region of maximal involvement: if any one of the 8 regions was considered abnormal, i.e., any regional mCERAD_SOT_ was > 1.5, the whole brain was considered abnormal. Conversely, only if all regions were considered normal (≤1.5) was the brain considered β-amyloid normal (see Table 1). The dichotomy of the brain as normal or abnormal was used for sensistivity and specificity calculations mandated as an endpoint for the trial whereas the continuous variable mCERAD_SOT_ provided a less discrete measure to compare to SUVR.

For mCERAD_mode_, the 30 measures made in each region (middle frontal, superior and middle temporal, and inferior parietal regions only) were averaged using mode to provide 4 regional categorical measures of *none*, *sparse*, *moderate*, or *frequent*. The brain was then dichotomised as abnormal if any region had a categorical assessment of *moderate* or *frequent* (see Table 1).

All measures were performed at a single laboratory to ensure consistency within the cohort as a consensus by two neuropathologists (JI and CS) against *a priori* criteria and blinded to clinical and imaging data and to the results of other histopathology analyses.

### β-amyloid immunohistochemistry

Immunohistochemistry for β-amyloid was a secondary reference standard used for two sets of analyses (correlation with PET tracer retention measurements and neuropathology diagnoses). β-amyloid IHC (monoclonal antibody, clone 4G8; SIG-39220, Covance, USA, diluted 1:100) was performed on 5 μm sections after 88% (v/v) formic acid pre-treatment for 5 min and heat-mediated antigen retrieval and detected using biotinylated secondary antibody (DakoCytomation E0354, UK) and DABMap Kit (Ventana, USA). Staining was pre-validated and standardized on an automated Ventana Discovery XT staining module.

In the first 30 brains analysed, the percentage of grey-matter area stained by the monoclonal antibody 4G8 was used for correlation analysis with regional PET SUVR measures. This analysis was performed on 3 step sections (separated by 100 μm) from the anterior block of each cortical region. For each of 5 randomly selected grey matter 100x fields of view per section, automated histometric measures of the % area of β-amyloid in 4G8-stained sections were recorded. A mean value was determined for each cortical region. Stained sections were imaged using whole slide scanning (Aperio XT) with a pre-developed and validated macro (MATLAB; MathWorks Inc, MA, USA) used to threshold intensity, size, and morphometry after colour deconvolution to remove the haematoxylin staining channel. All histometric measures were performed at a single laboratory to ensure consistency within the cohort by individuals who were blinded to clinical and imaging data and to the results of other histopathology analyses.

### Neuropathology diagnoses

For central laboratory neuropathological diagnosis, single sections from each of the 8 cortical regions and 11 additional neuropathology regions listed above were stained with haematoxylin and eosin, Bielschowsky silver (as above), β-amyloid IHC (as above), and Tau IHC (monoclonal antibody AT8; Cat No. MN1020, Thermo Scientific, UK). Additional sections from the entorhinal cortex and midbrain were stained for α-synuclein (monoclonal antibody; NCL-L-ASYN, Leica Microsystems, U.K.) to assess Lewy body pathology, and ubiquitin (polyclonal antibody; Z0458, DakoCytomation, UK). Neuropathology reports were prepared, including macroscopic observations, microscopic findings and relevant interpretive diagnoses. Braak staging of neurofibrillary tangles (NFT) [8], CERAD scoring of neuritic plaque frequency [43], β-amyloid phase analysis [58], and AD likelihood according to the National Institute of Aging-Reagan Institute (NIA-RI) diagnostic criteria [26] were recorded on case report forms. Where additional analyses were reported by the referring sites (e.g. TDP-43 immunopositivity), these were transferred to the case report forms. Neuropathology diagnoses were made at a single centre by two neuropathologists (AC and AI) to ensure consistency across the cohort. A diagnosis of AD was made according to the National Institutes of Ageing – Reagan Institute criteria [9]. Both neuropathologists were blinded to clinical and imaging data and to the results of cortical neuritic plaque analyses. Because of blinding to clinical data, neuropathology diagnoses were made with the assumption of dementia. AD neuropathologic changes were also categorised *post-hoc* according to the more recent NIA-Alzheimer’s Association criteria (NIA-AA) [25] using amyloid phase [58], Braak NFT stage and CERAD neuritic plaque score, collected above, as these criteria were published after trial initiation. Cerebral amyloid angiopathy (CAA) was categorised by Vonsattel grade, stage and type [57]. Other diagnoses were made against the following criteria; Tangle predominant dementia [68, 29], frontotemporal lobar degeneration (Picks Disease) [29], dementia with Lewy bodies [42], multiple system atrophy [22], and progressive supranuclear palsy [23, 40]. It should be noted that subsequent to the trial additional guidelines and diagnostic criteria for AD [25] and primary age-related tauopathy [13] have been published.

### PET imaging and analysis

The acquisition of [^18^F]flutemetamol PET images and analysis are described in detail elsewhere [14]. Briefly, Flutemetamol F 18 Injection was administered intravenously at a dose of 185-370 MBq of radioactivity at physician discretion, based on how long a scan the patient could be anticipated to tolerate; scan times were adjusted to dose to ensure equivalent scan quality with minimal patient discomfort. PET images were acquired in 2-min frames on PET/computerized tomography (CT) scanners, beginning approximately 90 min post injection. Typically, for an injected activity of 370 MBq, five 2-min frames were summed to give a 10-min scan, which was attenuation-corrected using CT data. Most images were reconstructed iteratively to form axial slices of 128 X 128 pixels and with ~90 slices covering the entire cerebrum. Image reconstructions were optimised at each PET centre using a NEMA IEC phantom [46] prior to patient imaging. In addition, a CT-scan was acquired. Equipment used to capture images varied across the study sites. The images were evaluated overall as either positive (abnormal/pathological) or negative (normal) for fibrillar β-amyloid in a blinded image evaluation (BIE) by 5 independent image readers who were blinded to all clinical, demographic, and pathology information. Readers were trained independently using GE Healthcare’s automated reader training program [17, 54, 63]. The interactive training program gave instruction on brain anatomy, image display, and assessment methodology to classify 5 regions; frontal lobe, lateral temporal lobe, parietal lobe, posterior cingulate/precuneus, and the striatum as either normal or abnormal. Following the training, readers had to pass a test in which they had to correctly classify at least 14 out of 15 test cases as normal or abnormal. Five trained readers classified the study cases as normal or abnormal using the same reading and classifying methodology. If any one of the regions assessed in a given case image was deemed to be abnormal, the case was dichotomised as a positive image assessment [49]. The confidence with which a reader was able to reach the decision made was recorded on a scale of 1–5 (5 being most confident). The majority read for each subject’s PET image is defined as the image interpretation (normal/abnormal) made by at least 3 of the 5 readers. BIE analysis of the first 68 subjects (Study GE067-007) by 5 different readers from those used in GE067-026 are presented elsewhere [14] and are not part of this *post-hoc* analysis. It should be noted that PET assessments in this study was based solely upon image assessment by readers, and that SUVR measures (see below) were not used for primary dichotomous assessment as normal or abnormal.

### Mapping histopathology measures to quantitative PET image SUVR measures

A detailed mapping regimen was implemented for the first 30 brains to co-register SUVR measurements with the 8 neocortical samples. SUVRs were measures of neocortical PET tracer retention normalised to the retention in the relatively spared cerebellum (SUVR_cer_) or pons (SUVR_pon_). Individual regional values were recorded and a composite SUVR (average across the 8 neocortical regions) was calculated. These measures were used as a pre-planned analysis to correlate tracer retention with β-amyloid histopathology as continuous rather than dichotomised variables. In addition to the first 30 brains, 2 further brains were added to this analysis *post-hoc* to understand the underlying pathology of false positives (cases 29 and 43 in Table 2). Photographs of the left hemispheres were taken pre- and post-sampling during the recording of macroscopic observations by the neuropathologist and during dissection of the fixed brains (Additional file 1). These included reconstructed hemispheres post-sampling to record the precise anatomical location from which the histological measures were made. These images were uploaded to the trial image repository and used to identify the regions sampled on ante-mortem anatomic images (CT or MRI) allowing registration with the PET images and enabling the relevant volumes of interest (VOIs) to be identified and to determine the regional SUVR. To ensure consistency across all subjects, this mapping was performed by a single individual (KH) who was blinded to clinical data and histopathology.

**Table 2:** Subject demographic information and data

Demographics					Neuropathology							Imaging	
Case	Age	Sex	Time^a^	Dementia^b^	mCs^c^	Aβ^d^	AD^e^	CERAD^f^	Braak^g^	Amyloid phase^h^	Diagnoses^i^	SUVR^j^	PETmaj^k^
1	73	F	360	Yes	0.0	n.d.	Normal	-	II	1	Inf TDP^l^	1.13	Normal
2	84	M	17	Yes	0.0	0.3	Normal	-	I	0	Normal	1.18	Normal
3	83	M	568	No	0.0	n.d.	Normal	-	IV	1	LBD	1.17	Normal
4	91	M	130	Yes	0.0	0.9	Normal	-	0	0	PSP VAD	1.34	Normal
5	63	M	433	No	0.0	n.d.	Low	-	II	1	PSP	1.42	Normal
6	76	F	145	Yes	0.0	n.d.	Normal	-	II	1	LBD VAD	1.22	Normal
7	70	M	16	No	0.0	n.d.	Normal	-	0	0	Normal	1.44	Normal
8	67	M	32	No	0.0	n.d.	Normal	-	I	0	Normal	1.37	Normal
9	80	M	131	Yes	0.0	n.d.	NA	-	III	0	TPD	1.26	Normal
10	61	F	34	No	0.0	n.d.	Normal	-	0	1	Normal	1.67	Normal
11	65	F	393	No	0.0	n.d.	Normal	-	III	1	AC	1.21	Normal
12	60	M	374	No	0.0	n.d.	Low	-	III	1	VAD	1.34	Normal
13	74	M	170	Yes	0.1	8.1	Normal	-	0	2	VAD	1.12	Normal
14	66	M	155	No	0.1	n.d.	Normal	-	0	0	AC	1.3	Normal
15	76	F	10	Yes	0.1	0.2	Normal	-	I	2	Normal	1.56	Normal
16	63	M	12	No	0.1	n.d.	Normal	-	0	0	Normal	1.6	Normal
17	73	F	105	Yes	0.5	0.4	Normal	-	V	1	TPD	1.34	Normal
18	90	F	115	Yes	0.0	n.d.	Low	S	III	1	Inf AS	1.4	Normal
19	89	F	78	No	0.3	n.d.	Int	S	IV	2	CAA AD	1.25	Normal
20	82	F	24	Yes	0.4	0	Low	S	III	1	LBD	1.72	Normal
21	92	F	210	Yes	0.7	n.d.	Normal	S	II	4	CAA PD	1.55	Normal
22	84	F	69	Yes	1.1	1	Int	S	II	2	Inf	1.36	Normal
23	72	M	142	Yes	1.3	n.d.	Normal	S	0	3	FTD	1.01	Normal
24	87	F	76	Yes	1.4	2	Low	S	I	3	VAD	1.57	Normal
25	87	F	137	Yes	1.5	n.d.	Normal	S	0	2	AC AS	1.53	Normal
26	60	M	11	Yes	1.7	n.d.	Low	S	II	4	CAA	1.08	Normal
27	81	M	189	No	1.8	n.d.	Normal	S	I	4	ND	1.6	Normal
28	92	F	212	Yes	2.1	n.d.	Int	S	III	3	TDP^l^	1.26	Normal
29	87	F	131	Yes	1.4	8.1	Low	S	IV	5	LBD	1.95	Abnormal
30	96	F	630	Yes	1.9	n.d.	High	S	VI	5	AD	2.15	Abnormal
31	92	F	132	Yes	1.9	n.d.	Low	S	III	4	LBD	2.72	Abnormal
32	89	F	311	Yes	2.0	n.d.	Int	S	III	5	AD CAA VAD	2.33	Abnormal
33	88	F	118	Yes	2.1	n.d.	Low	S	II	4	Inf LBD AS	3.14	Abnormal
34	80	M	2	Yes	2.1	7.6	High	S	VI	5	AD LBD	2.1	Abnormal
35	94	F	19	Yes	2.1	7.7	Int	S	III	5	AD	1.95	Abnormal
36	88	F	329	Yes	2.1	n.d.	High	S	V	5	AD	2.84	Abnormal
37	74	F	550	Yes	2.8	n.d.	High	S	VI	5	AD	2.23	Abnormal
38	86	M	19	No	1.4	2.3	Int	M	III	3	AD	1.45	Normal
39	75	M	64	Yes	1.4	1.4	Int	M	II	3	Inf LBD	1.23	Normal
40	84	M	349	Yes	1.6	n.d.	Int	M	V	3	AD LBD AS VAD Ath	1.73	Normal
41	93	M	323	No	1.9	n.d.	Low	M	II	3	LBD	1.36	Normal
42	87	M	22	No	2.7	4	Int	M	IV	4	AD	2.04	Normal
43	86	F	193	Yes	0.3	9.4	Low	M	III	4	LBD	2.07	Abnormal
44	76	M	84	Yes	1.5	10.3	Low	M	II	3	LBD	1.87	Abnormal
45	75	M	373	Yes	1.6	n.d.	Int	M	IV	5	AD CAA	1.48	Abnormal
46	82	F	127	Yes	1.7	n.d.	Int	M	IV	4	AD LBD	2.32	Abnormal
47	86	M	395	Yes	1.8	n.d.	High	M	V	5	AD	2.83	Abnormal
48	93	F	500	Yes	1.8	n.d.	Int	M	V	3	AD AS VAD	1.6	Abnormal
49	84	M	45	Yes	1.8	n.d.	Low	M	II	3	VAD	1.85	Abnormal
50	93	F	243	Yes	1.9	n.d.	High	M	VI	5	AD	2.72	Abnormal
51	93	F	755	Yes	2.0	n.d.	High	M	IV	5	AD	2.2	Abnormal
52	80	M	276	Yes	2.0	n.d.	High	M	VI	4	AD	2.33	Abnormal
53	90	M	308	Yes	2.1	n.d.	Low	M	II	4	LBD	2.19	Abnormal
54	78	M	62	Yes	2.1	10.1	High	M	VI	5	AD LBD	2.86	Abnormal
55	86	F	747	Yes	2.1	n.d.	Int	M	IV	3	AD CAA	1.81	Abnormal
56	73	F	295	No	2.2	n.d.	Low	M	I	3	LBD	1.76	Abnormal
57	87	F	318	Yes	2.2	n.d.	Int	M	III	5	AD AS VAD Ath	2.26	Abnormal
58	88	F	266	Yes	2.2	n.d.	Int	M	III	4	AD LBD	1.91	Abnormal
59	88	F	79	Yes	2.3	17.6	High	M	VI	5	AD	1.67	Abnormal
60	93	F	396	Yes	2.3	n.d.	High	M	VI	5	AD CAA Inf	3.08	Abnormal
61	85	M	60	No	2.4	14.7	High	M	VI	5	CAA AD VAD	2.81	Abnormal
62	91	M	30	Yes	2.4	2.9	High	M	V	4	AD	1.86	Abnormal
63	95	F	15	Yes	2.4	7.5	High	M	VI	5	AD	1.89	Abnormal
64	79	M	42	No	2.4	n.d.	Int	M	III	4	CAA mCa AD	2.44	Abnormal
65	81	F	184	Yes	2.5	n.d.	Int	M	IV	3	LBD	1.66	Abnormal
66	84	F	193	Yes	2.6	n.d.	High	M	V	5	AD	2.4	Abnormal
67	72	M	268	Yes	2.7	n.d.	High	M	VI	5	AD VAD	2.07	Abnormal
68	63	M	342	Yes	2.8	n.d.	High	M	VI	5	AD AS VAD	1.94	Abnormal
69	89	F	115	Yes	3.0	n.d.	High	M	VI	5	AD CAA LBD AS	2.39	Abnormal
70	83	F	611	Yes	1.8	n.d.	Low	F	I	4	AS CAA VAD TDP^l^	1.87	Normal
71	84	F	189	No	1.7	n.d.	Int	F	0	3	CAA Inf VAD AD	2.02	Abnormal
72	82	M	397	Yes	1.9	n.d.	High	F	VI	5	AD CAA VAD	2.41	Abnormal
73	86	F	155	Yes	2.0	n.d.	High	F	V	5	AD	2.43	Abnormal
74	93	F	594	Yes	2.0	n.d.	High	F	IV	5	AD	2.46	Abnormal
75	90	F	538	Yes	2.0	n.d.	High	F	VI	4	AD CAA AS VAD	2.78	Abnormal
76	78	F	180	Yes	2.2	n.d.	Normal	F	0	4	MSA	2.03	Abnormal
77	93	F	200	Yes	2.2	n.d.	High	F	V	5	AD AS CAA LBD VAD	2.42	Abnormal
78	78	F	125	Yes	2.2	n.d.	High	F	VI	5	AD LBD	2.12	Abnormal
79	72	M	1	Yes	2.4	11.4	High	F	VI	5	AD	2.37	Abnormal
80	76	F	27	Yes	2.4	n.d.	High	F	VI	5	AD CAA	1.83	Abnormal
81	77	F	11	Yes	2.4	8.9	High	F	VI	4	AD	1.59	Abnormal
82	91	F	55	Yes	2.4	11.2	High	F	VI	4	AD CAA	2.2	Abnormal
83	81	M	204	Yes	2.4	n.d.	High	F	VI	4	AD CAA LBD	2.41	Abnormal
84	82	M	15	Yes	2.5	6.9	High	F	VI	4	AD CAA	2.23	Abnormal
85	83	M	34	Yes	2.5	8.5	High	F	VI	5	AD	2.6	Abnormal
86	90	F	51	Yes	2.5	12.2	High	F	VI	4	AD	2.35	Abnormal
87	73	F	27	Yes	2.5	9.6	High	F	VI	5	AD	2.23	Abnormal
88	87	M	1	Yes	2.5	7.8	High	F	IV	4	AD CAA	2.1	Abnormal
89	89	F	768	Yes	2.5	n.d.	High	F	V	5	AD CAA LBD AS	1.93	Abnormal
90	79	M	332	Yes	2.5	n.d.	High	F	VI	5	AD CAA LBD	2.24	Abnormal
91	80	M	0	Yes	2.6	7.9	High	F	VI	5	AD	2	Abnormal
92	79	F	422	Yes	2.6	n.d.	High	F	V	5	AD CAA LBD AS VAD HC	2.38	Abnormal
93	87	M	106	Yes	2.6	n.d.	High	F	VI	5	AD	2.2	Abnormal
94	66	F	139	Yes	2.7	n.d.	High	F	VI	5	AD	2.37	Abnormal
95	84	M	181	Yes	2.7	n.d.	High	F	V	4	AD LBD	2.75	Abnormal
96	87	M	769	Yes	2.7	n.d.	High	F	VI	5	AD CAA LBD VAD	2.5	Abnormal
97	71	M	305	Yes	2.7	n.d.	High	F	V	5	AD CAA	2.47	Abnormal
98	72	F	565	Yes	2.7	n.d.	High	F	VI	5	AD CAA LBD	2.58	Abnormal
99	85	M	846	Yes	2.8	n.d.	High	F	VI	5	AD VAD	2.01	Abnormal
100	84	F	198	Yes	2.8	6.3	High	F	VI	4	AD	1.48	Abnormal
101	85	F	436	Yes	2.9	n.d.	High	F	VI	5	AD	2.65	Abnormal
102	75	F	66	Yes	2.9	10.6	High	F	VI	5	AD	2.47	Abnormal
103	87	M	493	No	2.9	n.d.	High	F	IV	5	CAA LBD AD	2.42	Abnormal
104	86	F	127	Yes	3.0	n.d.	High	F	VI	5	AD	2.9	Abnormal
105	81	M	171	Yes	3.0	n.d.	High	F	VI	5	AD	2.34	Abnormal
106	63	M	562	Yes	3.0	n.d.	High	F	VI	5	AD	2.72	Abnormal

Subjects are ranked by CERAD neuritic plaque frequency and then by mCERAD_SOT_. *AC* ageing changes, *AD* Alzheimer’s disease (high or intermediate likelihood by National Institute of Ageing-Reagan Institute criteria), *AS* arteriosclerosis or arteriolosclerosis, *Ath* atherosclerosis, *CAA* cerebral amyloid angiopathy, *FTD* frontotemporal lobar degeneration, *HC* hydrocephalus, *Inf* infarct, *LBD* Lewy body disease, *mCa* metastatic carcinoma, *MSA* multisystem atrophy, *ND* neurofibrillary degeneration, *PD* Parkinson’s disease, *PSP* progressive supranuclear palsy, *SUVR* standard retention value ratio, *TDP+* TDP43 immunopositivity, *TPD* tangle-predominant dementia, *VSD* vascular disease not otherwise specified

^a^Time between PET imaging and death in days

^b^Dementia recorded in the study medical history

^c^mCERAD_SOT_; maximal regional mean score determining Standard of Truth assignation as abnormal if > 1.5

^d^Percentage area of grey matter stained positively by amyloid β immunohistochemistry (4G8) determined only on a subset (32 subjects) of the cohort

^e^Alzheimer’s Disease likelihood recorded by a neuropathologist against National Institute of Ageing-Reagan Institute criteria [26] but blinded to dementia status

^f^CERAD neuritic plaque frequency recorded during neuropathology diagnoses (N: none; S: sparse; M: moderate; F: frequent)

^g^Braak neurofibrillary tangle stage recorded during neuropathology diagnosis

^h^Amyloid phase [25, 58]

^i^Neuropathologist’s diagnoses blinded to clinical data. Note: co-incident plaque burden may be present in non-AD diagnoses

^j^Composite SUVR determined from bilateral measures and normalised to cerebellum as the reference region

^k^Majority PET image evaluation

^l^TDP43 immunopositivity was recorded at the site neuropathology laboratories, not as part of the diagnoses for the GE studies. This analysis was not performed on all subjects

### Data collection and analysis

All data were collected from the clinical, imaging and pathology analysis centres via case report forms, which were quality-controlled before being transferred to a central data management centre (i3 Statprobe, Austin, Texas, USA, for trial GE067-007 and H2O Clinical LLC, Hunt Valley, Maryland, USA for GE067-026). Pathology data from the initial 68 brains in trial GE067-007 were used verbatim for the extended GE067-026 trial and no reanalysis was performed on these brains. All data blinds were maintained until the database was locked, at which point the data for the study primary objectives were analysed. The study GE067-026 [49] met its pre-defined success criteria of the lower bound of the 95% exact binomial confidence interval exceeding 75% for sensitivity and 60% for specificity for the majority interpretation. All analyses presented here represent *post-hoc* analyses of pathology data collected during the clinical trials GE067-007 (*N* = 68) and GE067-026 (*N* = 106), regional co-registered histopathology correlates and SUVRs collected in a subset (*N* = 32) of the GE067-007 subjects, and PET image evaluation by 5 independent readers of all 106 subjects read as a single trial GE067-026.

**Fig. 1:**
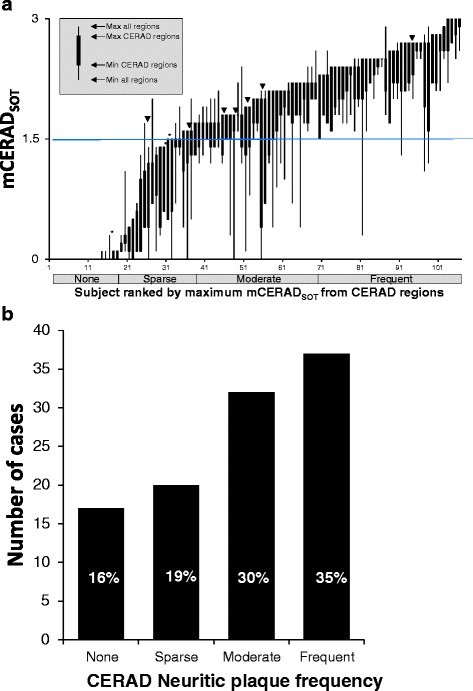
The GE067-026 cohort contained a broad and continuous spectrum of neocortical neuritic plaque burden. Panel **a**. Subjects ranked by the maximal mCERAD_SOT_ score for CERAD regions demonstrating a number of cases where dichotomy was based on a burden close to the threshold of 1.5. (cases were determined to be abnormal if any regional score was greater than 1.5, i.e. if the upper ‘whiskers’ in the plot are over 1.5). Most disparities between pathology and PET dichotomy as abnormal or normal occur in cases where the neuritic plaque mCERAD_SOT_ score is close to the threshold. Downward arrowheads indicate abnormal cases that were assessed as normal by PET (false negatives) and asterisks indicate normal cases assessed as abnormal (false positives). Panel **b**. The spread of cases categorised by CERAD neuritic plaque frequency

**Fig. 2:**
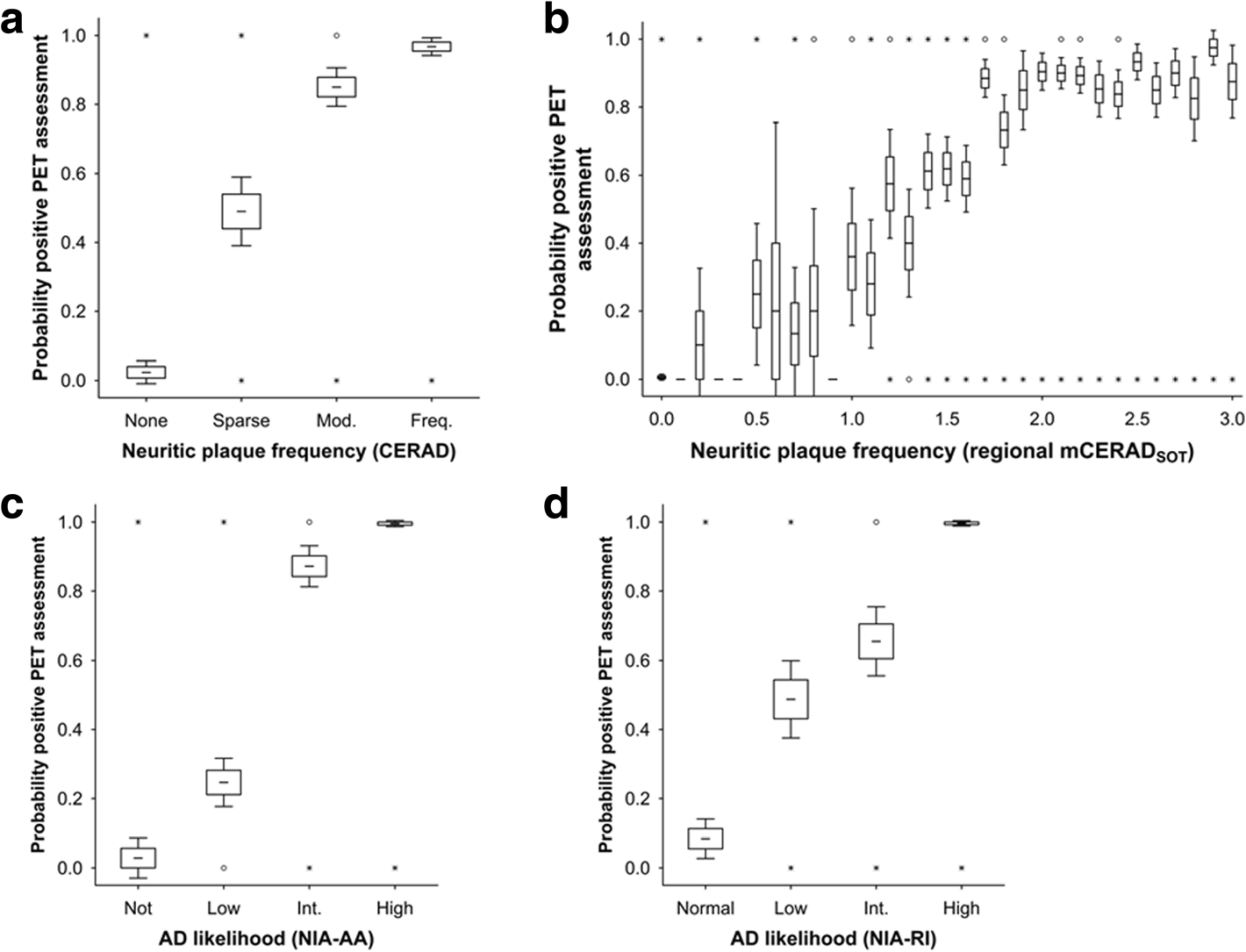
The probability that the PET image is interpreted as positive increases with cortical neuritic plaque burden. Panel **a** *.* The probability (0-1) of a PET positive assessment increases with CERAD neuritic plaque frequency (*N* = 106). Panel **b** *.* The probability of PET positive image assessment by neocortical regional mCERAD_SOT_ score (*N* = 424, 4 neocortical regions per case; frontal, temporal, parietal and posterior cingulate/precuneus). Panel **c** & **d**. The probability of PET positive image assessment increases by AD diagnosis against the NIA-AA (Panel **c**) and NIA-RI criteria (Panel **d**). For all panels, boxes represent mean +/- 1 standard error and whiskers represent 95% confidence interval. Open circles represent outlier values and asterisks represent extreme values (see Materials and methods for details)

**Fig. 3:**
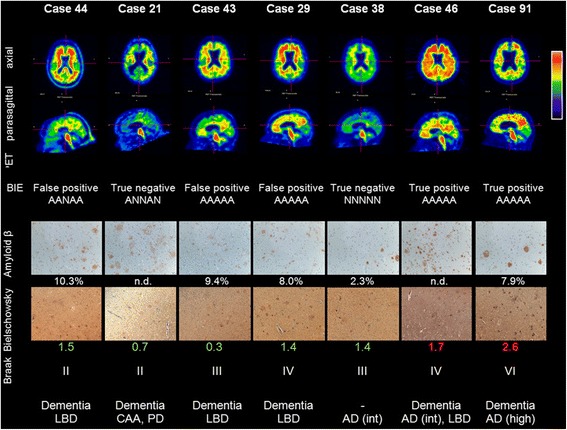
PET images and representative histopathology for a range of subjects including some disparity cases (PET images are representative Rainbow colour scale, axial and parasagittal slices. BIE status and results for the 5 readers (*N* = normal, A = abnormal). A representative photomicrograph of β-amyloid IHC (frontal lobe) with % area (if determined), representative photomicrograph of Bielschowsky silver stain (frontal lobe) and Bielschowsky score (original magnification 100x for both); Braak stage of neurofibrillary tangles and overall neuropathological diagnosis, including AD likelihood (NIA-RI criteria). Note: the photomicrographs of the frontal lobe may not accurately represent the pathology of other regions

**Fig. 4:**
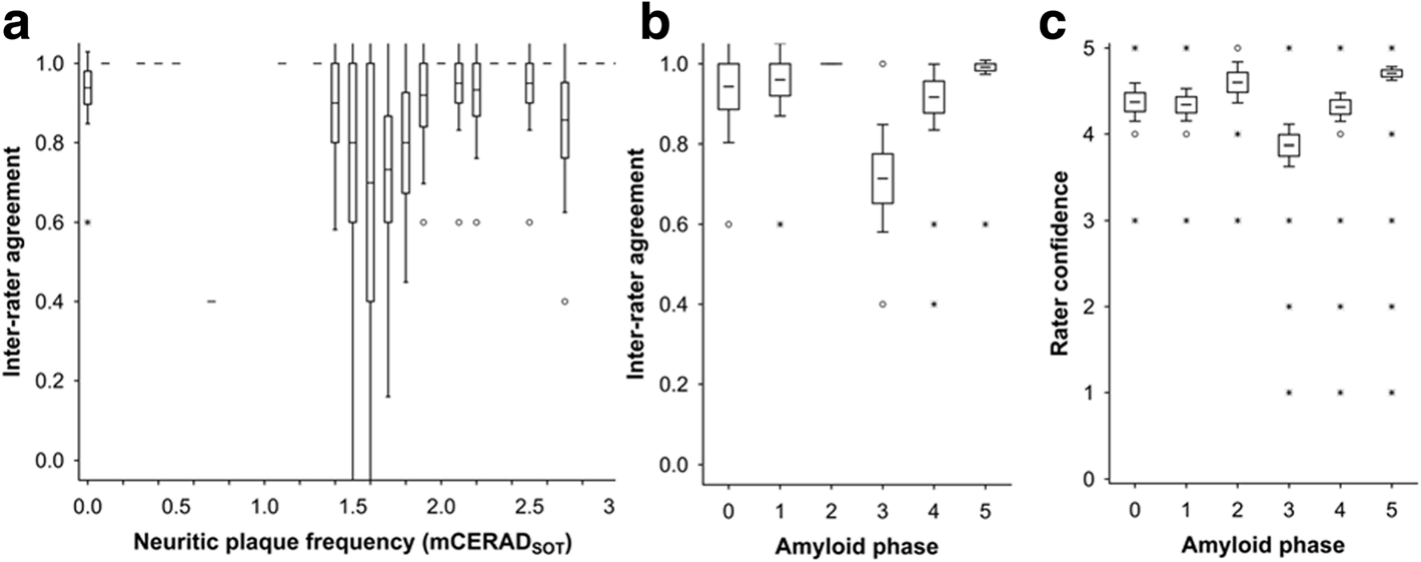
Inter-reader agreement and reader confidence are decreased in cases with a plaque burden close to the threshold of PET detection. Panel **a** shows inter-reader agreement across the spectrum of mCERAD_SOT_ scores with values lowest about the 1.5 threshold. The transition from amyloid phase 2 to phase 3 is associated with neocortical plaque burden between sparse and moderate (see Fig. 4) and both inter-reader agreement (Panel **b**) and reader confidence (Panel **c**) is lowest in phase 3 (*P* < 0.001 for both data sets, Kruskal Wallis test). Inter-reader agreement was determined as Fleiss’ kappa coefficient *P*_*i*_ (see text for details) and for 5 readers is 1 when all 5 readers are in agreement, 0.6 when the agreement is 4:1 and 0.4 when the agreement is split 3:2. Confidence was recorded as a 5 point scale (1–5) with 5 being most certain and 1 being the least certain. For all panels, boxes represent mean +/- 1 standard error and whiskers represent 95% confidence interval. Open circles represent outlier values and asterisks represent extreme values (see Materials and methods for details)

**Fig. 5:**
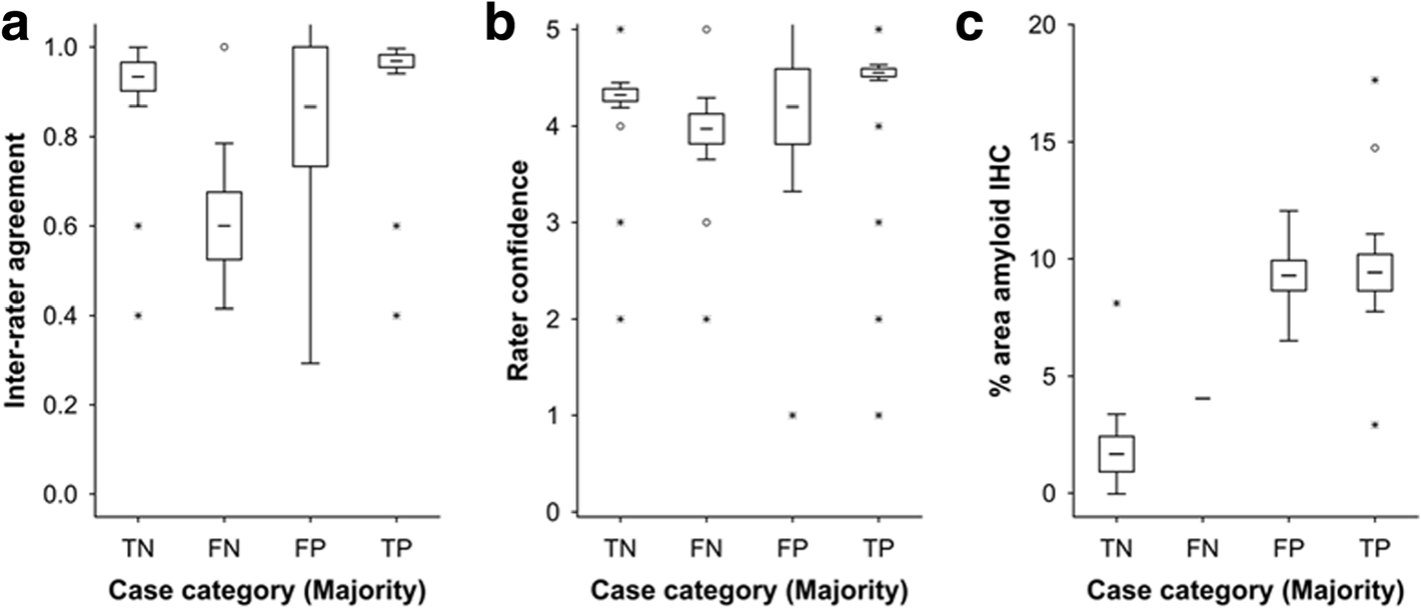
Cases in which there was disparity between the pathology dichotomy as normal or abnormal and PET negative or positive were associated with low reader confidence (Panel **a**) and inter-reader agreement (Panel **b**). False-negative cases (FN) where the majority interpreted the PET image as negative in the presence of an abnormal neuritic plaque burden were associated with low inter-reader agreement (*P* < 0.0001, Kruskal Wallis test). False-positive (FP) cases were associated with low reader confidence (*P* < 0.0001, Kruskal Wallis test). Inter-reader agreement was determined as Fleiss’ kappa coefficient *P*_*i*_ (see text for details) which for 5 readers is 1 when all 5 readers are in agreement, 0.6 when the agreement is 4:1 and 0.4 when the agreement is split 3:2. Reader confidence was recorded as a 5-point scale (1-5) with 5 being most certain and 1 being the least certain. Panel **c** In the 3 false-positive cases, although neuritic plaque frequency was below threshold, β-amyloid in the form of diffuse plaques was comparable to mCERAD_SOT_ cases (true positives; TP). One case also had high % area stained for β-amyloid by IHC but was low intensity and the readers called this case true negative (TN) (reader ratio 5:0, Case 13) suggesting that in this case cortical β-amyloid levels in the absence of any neuritic plaques were insufficient to produce a positive image by PET. Another case had sufficient neuritic plaques (mCERAD_SOT_ 2.7) even though the % area stained was relatively low. For all panels, boxes represent mean +/- 1 standard error and whiskers represent 95% confidence interval. Open circles represent outlier values and asterisks represent extreme values (see Materials and methods for details)

**Fig. 6:**
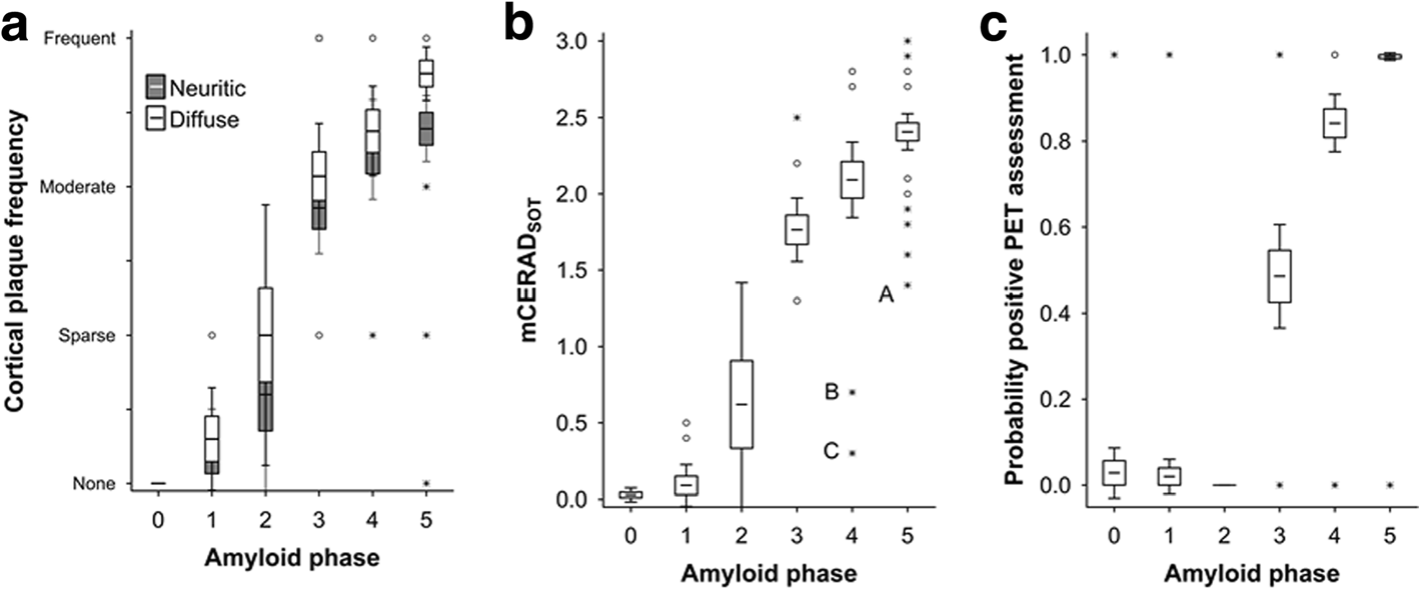
Neocortical diffuse and neuritic plaque frequency and probability of PET positive interpretation increases with β-amyloid phase. Amyloid phase represents a progression of plaque deposition with advancing AD starting in the neocortex (phase 0-2) and progressing into the midbrain (phase 3) and hindbrain (phase 5). By phase 4, neocortical neuritic plaques are sufficiently abundant to be detectable by PET imaging. Panel **a**. The abundance of both diffuse and neuritic plaques increase with advancing amyloid phase. The plots represent mean neocortical plaque frequencies (0 = none, 1 = sparse, 2 = moderate and 3 = frequent) per amyloid phase and plaque frequency is determined by the CERAD single point estimate for each subject (*N* = 106). Panel **b** *.* Neocortical plaque frequencies as determined by mCERAD_SOT_ using multiple measures per region (*N* = 106). Note three outliers (solid black circles) in phase 4/5 with mCERAD_SOT_ below 1.5 meaning that these cases were considered normal by mCERAD_SOT_ but abnormal by amyloid phase. Two of these cases (A and C) were false positives by majority, while B was true negative by majority, but only by a 3:2 reader split. Panel **c** *.* The probability of positive global PET assessment increases with amyloid phase (*N* = 5 for each subject, *N* = 35, 50, 25, 70, 120 and 230 for phases 0–5 respectively). For all panels, boxes represent mean +/- 1 standard error and whiskers represent 95% confidence interval. Open circles represent outlier values and asterisks represent extreme values (see Materials and methods for details)

**Fig. 7:**
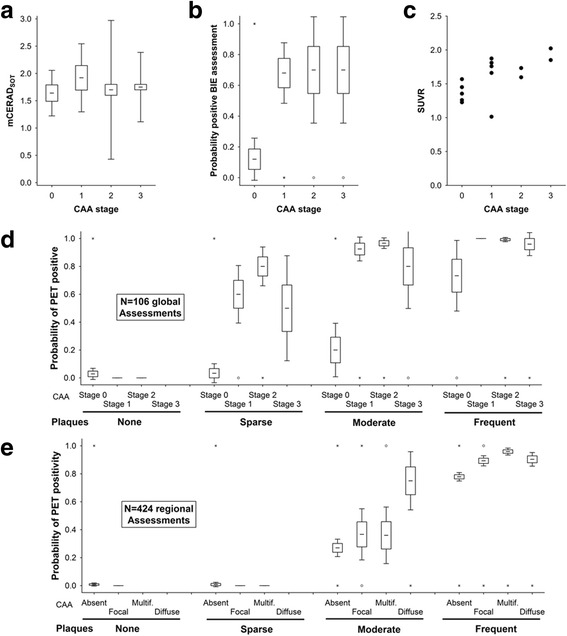
CAA contributes weakly to PET cortical positivity. In amyloid phase 3 cases, where cortical neuritic plaque load is borderline (Panel **a**), PET assessment as abnormal is more likely (*P* = 0.0001, Kuskal Wallis test) in the presence of CAA (Panel **b**) and SUVR is elevated (Panel **c**, *P* < 0.05 Spearman). Panel **d**. Probability of positive BIE assessment for all subjects demonstrating that this analysis is only possible in cases with a modest plaque burden (Moderate or phase 3 cases) because of a lack of amyloid angiopathy in phase 0–2 and certain cortical positivity due to abundant cortical neuritic plaques in Phase 4 and 5. Panel **e**. A similar pattern if observed with regional analysis (frontal, temporal, parietal and striatum). It is likely that this effect is subtle and additive to a neocortical plaque burden already close to the threshold. Boxes represent mean +/- 1 standard error and whiskers represent 95% confidence interval. Open circles represent outlier values and asterisks represent extreme values (see Materials and methods for details)

Receiver operating characteristic (ROC) analysis was used to determine optimal threshold values for the *post-hoc* analysis of regional mCERAD_SOT_, β-amyloid IHC % area measures, and for SUVR thresholds. SUVR comparisons were performed using parametric analysis of variance (ANOVA). The Fleiss coefficient P_i_ was used to measure inter-reader agreement [P_i_ = 1/n(n-1)*(A^2^ + N^2^-n), where n is the number of readers, A is the number calling the case abnormal, and N is the number of readers calling the case negative] [18]. Non-parametric tests for correlation (Spearman’s), differences (Wilcoxon Rank Sum Test or Mann-Whitney *U* test, Kruskal-Wallis test), and contingency analysis (*χ* ^2^ and Fisher’s Exact Test) were used for all other analyses where indicated. All statistical tests were performed using StatSoft Statistica software (Tulsa, USA) unless otherwise stated. All graphs and figures (Except Figs. 1 and 3) were also produced using Statistica software. Graphical representation in box plots represent mean +/- 1 standard error (SE, boxes) and 95% confidence intervals (whiskers). Outlier and extreme values are presented as open circles and asterisks, respectively and are identified by the Statistica software as; outlier values > UBV + o.c.*(UBV-LBV) OR < LBV - o.c.*(UBV • LBV); extreme values > UBV + 2*o.c.*(UBV-LBV) OR < LBV - 2* o.c.*(UBV • LBV) where UBV is the upper box value (mean + 1SE), LBV is the lower box value (mean-1SE) and o.c. is the outlier coefficient set at 1.5. Note for probability plots (Figs. 2a–d, 6c and 7b, d & e) all values are either 0 or 1 and extreme and outlier values will always be 0 or 1. For confidence plots (Figs. 4c and 5b) all values are integers between 1 and 5 and so all outlier and extreme values will be integers. For inter-reader agreement plots (Figs. 4a & b and 5a) P_i_ values are 1 (5/5 agreement), 0.6 (4:1 or 1:4 agreement) or 0.4 (3:2 or 2:3 agreement) and all outlier and extreme values will have these values. For all other plots outliers and extremes are continuous variables.

## Results

The GE067-026 cohort of 106 brains included a broad and continuous spectrum of β-amyloid pathology based on mCERAD_SOT_ and CERAD assessments (Fig. 1a). Seventy-six brains (72%) were determined to be abnormal by mCERAD_SOT_, and 30 (28%) were assessed as normal; together, these brains provided a continuous distribution of cases throughout the range of neuritic plaque pathology (Fig. 1b). Demographic data, pathology, and majority PET BIE assessments for all subjects are given in Table 2. Sixty-six cases had a neuropathological diagnosis of AD (62%), defined as either ‘intermediate’ or ‘high’ likelihood by NIA-RI criteria [26], in which neuritic plaques are nominally *moderate* or *frequent* in the neocortex. While AD was the predominant neuropathological diagnosis (Table 3), it was often recorded with coincident pathologies, such as CAA (*n* = 24) or other vascular disease (*n* = 27), or Lewy body disease (LBD, *n* = 16). Just less than half of the 66 AD cases had “pure” AD (*n* = 30). Lewy body disease was the next most predominant diagnosis (*n* = 29; 27%) and, in addition to the 16 cases with coincident AD pathology sufficient for a diagnosis of AD, many other cases had varying degrees of co-incident β-amyloid neuropathology (Table 2). Indeed, many of the subjects without a primary diagnosis of AD had a range of coincident AD pathology, potentially allowing assessment of PET imaging in a target population where underlying AD pathology may be coincident with other dementing disorders. This mixed pathology population included cases with neuritic plaque frequencies close to the dichotomous threshold, i.e. between *sparse* (normal) and *moderate* (abnormal) which was the presumed *a priori* lower limit of plaque burden detectable with [^18^F]flutemetamol.

**Table 3:** PET majority, AD and mCERAD_SOT_ assessment by disease category

Disease category	Number	Percent	PETmaj abnormal	# With coincident AD^a^	# mCERAD_SOT_ abnormal
Alzheimer's Disease^a^	66	(62%)	62	66	64
Lewy Body Disease^b^	29	(27%)	23	16	22
Cerebral Amyloid Angiopathy^c^	27	(25%)	23	24	25
Vascular^d^	21	(20%)	14	14	16
Arteriosclerosis^e^	13	(12%)	9	9	11
Infarct	7	(7%)	3	2	3
Normal	6	(6%)	0	0	0
Ageing Changes	3	(3%)	0	0	0
TDP43 immunopositive^f^	3	(3%)	0	0	2
Tangle predominant dementia	2	(2%)	0	0	0
Progressive Supranuclear Palsy	2	(2%)	0	0	0
Atherosclerosis	2	(2%)	1	2	2
Frontotemporal Dementia	1	(1%)	0	0	0
Metastatic carcinoma	1	(1%)	1	1	1
Hydrocephalus	1	(1%)	1	1	1
Multiple system atrophy	1	(1%)	1	0	1
Neurofibrillary degeneration	1	(1%)	0	0	1
Parkinson's Disease	1	(1%)	0	0	0

*AD* Alzheimer’s disease, *mCERAD*_*SOT*_ standard of truth by modified Consortium to Establish a Registry for Alzheimer’s Disease criteria, *N* Number of subjects, *PETmaj* majority read of positron emission tomography images, SOT standard of truth

All neuropathology diagnoses made blind to clinical data

^a^Includes intermediate or high likelihood by National Institute of Ageing-Reagan Institute criteria irrespective of dementia status

^b^Diagnosed as dementia with Lewy bodies blinded to clinical data

^c^Does not include focal cerebral amyloid angiopathy

^d^Includes: multifocal infarcts, microinfarcts, cerebral vascular disease, vascular brain injury and vascular dementia

^e^includes arteriosclerosis and arteriolosclerosis

^f^TDP43 immunopositivity was recorded at the site neuropathology laboratories, not as part of the diagnoses for the GE studies. This analysis was not performed on all subjects

Subjects classified as abnormal by mCERAD_SOT_ were generally older than those categorised as normal (*P* < 0.01, Mann-Whitney) and the average age of female subjects was slightly older than that of males (81.5 vs 78.5 years respectively; *P* < 0.001 Mann-Whitney). mCERAD_SOT_ was independent of gender (*P* = 0.57 Mann-Whitney). Most histometric measures of plaques, tangles, and diagnoses were correlated (Table 4), as would be expected, given that the diagnoses of AD were derived from these measures.

**Table 4:** Histometric, neuropathology, clinical and BIE correlates

mCERAD_SOT_	Amyloid phase	% Amyloid IHC	Dx AD (NIA-RI)	Dx AD (NIA-AA)	Braak stage	Cortical atrophy	SUVR	Dementia	BIE positivity		
(Quantitative)	(Ordinal)	(Quantitative)	(Ordinal)	(Ordinal)	(Ordinal)	(Ordinal)	(Quantitative)	(Nominal)	(Nominal)		
*P* < 0.0001	*P* < 0.0001	*P* = 0.0005	*P* < 0.0001	*P* < 0.0001	*P* < 0.0001	*P* = 0.2518	*P* < 0.0001	*P* = 0.0009	*P* < 0.0001	CERAD	(Ordinal)
	*P* < 0.0001	*P* = 0.0014	*P* < 0.0001	*P* < 0.0001	*P* < 0.0001	*P* = 0.3569	*P* < 0.0001	*P* = 0.0021	*P* < 0.0001	mCERAD_SOT_	(Quantitative)
		*P* < 0.0001	*P* < 0.0001	*P* < 0.0001	*P* < 0.0001	*P* = 0.6275	*P* < 0.0001	*P* < 0.0001	*P* < 0.0001	Amyloid Phase	(Ordinal)
			*P* = 0.0001	*P* < 0.0001	*P* = 0.0001	*P* = 0.9266	*P* < 0.0001	*P* = 0.8746	*P* < 0.0001	% amyloid IHC	(Quantitative)
				*P* < 0.0001	*P* < 0.0001	*P* = 0.2087	*P* < 0.0001	*P* < 0.0001	*P* < 0.0001	AD (NIA-RI)	(Ordinal)
					*P* < 0.0001	*P* = 0.2758	*P* < 0.0001	*P* < 0.0001	*P* < 0.0001	AD (NIA-AA)	(Ordinal)
						*P* = 0.4195	*P* < 0.0001	*P* < 0.0001	*P* < 0.0001	Braak Stage	(Ordinal)
							*P* = 0.1778	*P* = 0.4006	*P* = 0.7397	Cortical atrophy	(Ordinal)
								*P* = 0.0041	*P* < 0.0001	SUVR	(Quantitative)
									*P* < 0.0001	Dementia	(Nominal)

*mCERAD*_*SOT*_ modified CERAD Standard of Truth, *IHC* Immunohistochemistry, *Dx AD (NIA-RI)* Neuropathological diagnosis of AD likelihood using the National Institute of Ageing – Reagan Institute criteria [9], *Dx AD (NIA-AA)* Neuropathological diagnosis of AD likelihood using the National Institute of Ageing – Alzheimer’s Association criteria [8], *SUVR* Standard retention value ratio normalised to the cerebellar cortex, *BIE* Blinded Image evaluation of PET images, *CERAD* Consortium to Establish a Registry for Alzheimer’s Disease

Statistical tests were performed as indicated in the insert panel. mCERADSOT, % area amyloid IHC and SUVR values are continuous variables. All other variables are either categorical; CERAD (None, Sparse, Moderate, Frequent); Amyloid Phase (phases 0, 1, 2, 3, 4 and 5); Dx AD (NIA-RI) (Normal, Low, Intermediate or High - likelihood of AD), Dx AD (NIA-RI) (Not, Low, Intermediate or High – level of AD neuropathologic change), Braak Stage (Stage 0, I, II, III, IV, V and VI); Cortical atrophy (None, Mild, Moderate, Severe), or nominal; Dementia (Yes or No); BIE positivity (Normal Abnormal). All tests were performed using Spearman except BIE positivity vs Dementia which was perfomed using Chi-square test. Plots for some BIE positivity correlations are presented in Figs. 2 and 4

Time between PET scan and death did not significantly influence the outcome although we note that one false negative case died 611 days after PET. While it is possible that the amyloid burden increased during this period we are unable to confirm this.

### Blinded image evaluations of PET Images

Seventy-two cases were assessed as positive (abnormal) by majority read and the remaining 34 cases were normal by majority read (Table 2). As β-amyloid is believed to deposit in phases, starting with the neocortex and advancing to subcortical regions [58], the pattern of regional PET assessment was examined. Only one case was classified as abnormal on the basis of a single cortical region showing abnormal retention (case 49). All other cases assessed as positive by majority were positive in more than one neocortical region. With respect to cortical versus striatal results (with the potential to compare to β-amyloid phases [58]), 6 cases were cortical positive and striatal negative (cases 44, 48, 49, 55, 58 and 64) and no case was deemed majority positive solely on the basis of an abnormal striatal retention pattern. Of the 72 majority BIE positive cases, 55 (76%) were abnormal in all 5 regions assessed and thus clearly positive. The posterior cingulate/precuneus was deemed abnormal in all cases positive by majority BIE. The striatum was deemed abnormal in 66 of these 72 positive cases by majority BIE.

The probability of the PET image being rated as abnormal increased with neocortical neuritic plaque frequency and AD diagnosis (Fig. 2). Illustrations of PET images alongside representative histopathology for select case are shown in Fig. 3, including some cases with discordant pathology versus PET results, which are discussed later.

Reader confidence was higher in cases deemed abnormal by mCERAD_SOT_ than in those deemed β-amyloid normal (*P* = 0.000125, Mann Whitney *U* test), suggesting that readers felt more confident identifying abnormal retention patterns than the normal white-matter retention pattern. Unsurprisingly, reader confidence was highest in highly abnormal cases i.e. CERAD frequent or amyloid phase 5 cases (Fig. 4) and inter-reader agreement was highest in cases that were either clearly positive (phase 5/frequent) or negative (phase 0–2/none). When neocortical β-amyloid pathology was close to the threshold (*sparse*/*moderate* or amyloid phase 3) reader confidence and inter-reader agreement worsened (Fig. 4). Readers were less confident assessing cases that were subsequently classified as false negatives compared to true negatives or true positives (*P* < 0.05 and *P* < 0.001 respectively Mann-Whitney *U* test) and inter-reader agreement was also worse (Fig. 5; *P* < 0.001 Kruskal-Wallis). Despite the temporal cortex being the most involved of the cortical regions by pathology, the temporal cortex was scored as abnormal by PET the least frequently of the cortical regions assessed. Inter-reader agreement and probability of a positive BIE assessment were lowest in the temporal cortex and in the striatum, although these differences did not reach statistical significance (Kruskal Wallis; *P* > 0.05). Observed regional BIE assessments were significantly different from those expected based upon the regional mCERAD_SOT_ scores (*P* = 0.0079; *χ* ^2^ test) with the temporal lobe assessed as abnormal less frequently than expected, and the other three cortical regions scored as abnormal slightly more frequently than expected. Together, these results possibly reflect a slightly more difficult interpretation of the temporal lobes compared to the other regions because of the combination of depth of sulci and partial volume effects.

Asymmetry was an occasional feature in the PET images, but while there was a small potential to misclassify cases if the β-amyloid burden was above threshold in the hemisphere not sampled for histopathology assessment, asymmetry was usually focal and no images showed asymmetry in all regions assessed (global asymmetry). Furthermore, there were very few cases dichotomized as abnormal by either PET or pathology based on a single region (which would be the cases most susceptible to misclassification), and so the risk of misclassification appeared small. Nevertheless, all cases where there was notable asymmetry (as determined post-hoc) were assessed for this potential. A potential to misclassify was only present under two circumstances. First, if pathology was normal in the left hemisphere but the PET image was assessed as abnormal, this could be incorrectly classified as a false positive if the pathologically unassessed right hemisphere was the basis for calling the image positive. However, there were no subjects with this combination. Second, if pathology (based on the left hemisphere) and PET assessments (based on both hemispheres) were both negative but there was β-amyloid pathology in the right hemisphere, then the case might have been wrongly categorized as a true negative when it would actually have been a false negative. Of five cases meeting this criterion, 4 showed minimal β-amyloid pathology; mCERAD_SOT_ values were 0, 0, 0 and 0.4, and all readers recorded negative PET assessments for all regions. One subject (case 39) did have left-sided cortical β-amyloid close to the threshold, but again all readers uniformly recorded negative cortex assessments from the bilateral [^18^F]flutemetamol image. The literature also suggests that there is general bilateral symmetry for β-amyloid by both PET [48] and pathology [44].

### Amyloid in neuritic plaques is the predominant β-amyloid pathology imaged by PET

The assessment of neuritic plaques in neocortical regions of the brains has been a key feature for the diagnosis of AD and was expected to be the predominant histopathological feature correlating with [^18^F]flutemetamol PET image assessment. CERAD, mCERAD_SOT_, and mCERAD_mode_ neuritic plaque assessments generally agreed, although the single-point estimate CERAD included disparities with mCERAD_SOT_ in 15/106 cases (14%) and was a less robust predictor of PET image positivity than both mCERAD_SOT_ and mCERAD_mode_ (Table 5). Four cases (4%) were dichotomised differently when using mCERAD_SOT_ vs. mCERAD_mode_. Significant plaque burdens in non-CERAD regions led to the differential classification of only two cases (cases 26 and 74), supporting the general view that β-amyloid pathology limited to non-CERAD regions (anterior cingulate and primary visual cortex in these cases) is atypical (2/106 = 2%), but should not be ignored when comparing to global cortical assessments used in β-amyloid PET imaging. Two further cases were dichotomised differently due to the statistical method used (mean vs. mode) in subjects with borderline β-amyloid pathology (cases 32 and 44). Indeed, discrepancies were only noted between any of the three neuritic plaque assessment methods when the burden was close to the threshold between sparse and moderate. Since these differences were so few in number and changed from false classifications (false negative, FN; false positive, FP) to true classifications (true negative, TN; true positive, TP) (and vice versa; case 26 FP → TN, case 32 TN → FN, case 44 FP → TP, and case 74 FN → TN; mCERAD_SOT_ → mCERAD_mode_ respectively), sensitivity and specificity of majority PET reads were comparable for the 2 methods (respectively 91% and 90% for mCERAD_SOT_ and 92% and 87.5% for mCERAD_mode_). These performance measures were superior to those using the single-point estimate CERAD assessment (91% and 76% for sensitivity and specificity, respectively).

**Table 5:** Summary statistics and case status for each of the β-amyloid dichotomy algorithms

	Case status (5 readers per case)				Pathology			
Reference standard	TN	FP	FN	TP	Normal	Abnormal	Sensitivity^a^	Specificity^b^
mCERAD_SOT_^c^	131	19	33	347	30	76	91%	87%
mCERAD_mode_^d^	134	26	30	340	32	74	92%	84%
CERAD^e^	134	51	30	315	37	69	91%	72%
Amyloid phase^f^	108	2	56	364	22	84	87%	98%

*TN* true negative, *FP* false positive, *FN* false negative, *TP* true positive

^a^sensitivity (true positive rate) = TP/(TP + FN)

^b^specificity (true negative rate) = TN/(TN + FP)

^c^Abnormal defined as any regional mCERAD_SOT_ score > 1.5). Note while these analyses are presented by individual reads, the *a priori* analysis was by majority read (sensitivity was 91% and specificity was 90% by majority read N = 106)

^d^Abnormal defined as any CERAD region moderate or frequent (multiple measures)

^e^Abnormal defined as and CERAD region moderate or frequent (single point estimate)

^f^Abnormal defined as phase 3, 4 or 5

Comparison of CERAD single-point estimates and mCERAD_SOT_ showed 15 disparate global assessments, all of which were in the *sparse* or *moderate* categories and close to the threshold between normal and abnormal. This supports the superiority of repeated measures over a single (or even duplicate) measure, but also highlights the potential for misclassification in any biological continuum when close to a fixed threshold. The fact that our SOT methods and the CERAD single point estimates all correlate well with PET tracer retention dichotomy strongly supports the notion that neuritic plaque burden is strongly associated with PET β-amyloid imaging. The probability of a subject having a positive PET image interpretation increased with neuritic plaque burden (Fig. 2a). Subjects with a *sparse* neuritic plaque frequency were equally as likely to have a positive or negative PET image, suggesting that the actual threshold of plaque burden that can be detected by [^18^F]flutemetamol (between sparse and moderate) is below the one generally accepted to be diagnostically relevant for AD diagnosis (moderate or frequent) [41]. Both cortical neuritic (Bielschowsky) and total (β-amyloid IHC) plaque burden was higher in the neocortex with advancing disease progression as determined by the β-amyloid phase [58] (Fig. 6) when measured as a single CERAD categorical point estimate or by mCERAD_SOT_ (Bielschowsky for neuritic plaques) or CERAD-like categorical score (β-amyloid IHC for diffuse plaques). This increase in cortical plaque burden was associated with an increase in the probability of positive PET assessment starting in phase 3 with near certainty in phase 5. The implication is that although neocortical plaques are present in phases 1 and 2, these are below the limit of detection by blinded visual assessment of [^18^F]flutemetamol PET images and that the threshold of a negative or positive PET image is reached between phase 3 and phase 4. These data also illustrate the increasing cortical β-amyloid burden associated with increasing subcortical β-amyloid distribution.

The maximal regional mCERAD_SOT_ score was observed most frequently in the middle temporal lobe in nearly half of all abnormal cases (Table 6). Other CERAD regions were also frequently observed to be the region of maximal involvement. While the precuneus was assessed as positive by PET in all BIE-abnormal cases, this region was only classified as abnormal by pathology in 75% of the cases globally classified as abnormal. Pathology in other regions was variable. β-amyloid burden in the primary visual cortex was highly variable. The maximal involvement of the CERAD regions is in broad agreement with accumulated histopathological studies that indicate a progressive spread of β-amyloid deposition within the brain, beginning in the lateral neocortex and basal neocortex [7] and spreading through medial neocortex, subcortical diencephalon, midbrain and then into the cerebellar and hindbrain structures [58]. These studies have also suggested that most neocortical regions have a heavy burden by the time the disease has progressed to symptomatic phases. While the frequencies of β-amyloid abnormality (mCERAD_SOT_ > 1.5) were greatest in CERAD regions (Table 6), there was no significant difference in mean mCERAD_SOT_ between the regions (*P* = 0.68; Kruskal-Wallis test *N* = 848). Of the 76 abnormal cases by mCERAD_SOT_, 40 (53%) were abnormal in all eight cortical regions and 70 (92%) were abnormal in multiple regions. Only 6 cases (8%) were dichotomised as abnormal on the basis of a single region and were therefore theoretically more prone to misclassification. Indeed, 3/6 of these single region dichotomies were discordant with PET (i.e. false-negative PET assessment by majority).

**Table 6:** Frequency of regional β-amyloid abnormality

	Neocortical region							
	CERAD regions							
	MFL	STG	MTG	IPL	ACG	PCG	PRC	PVC
Pathology								
Frequency region maximal^a^	13 (17%)	5 (7%)	33 (43%)	19 (25%)	8 (11%)	6 (8%)	12 (16%)	17 (22%)
Frequency region abnormal mCERAD_SOT_ >1.5 (of 76 abnormal cases)	61 (80%)	57 (75%)	65 (85%)	66 (87%)	56 (74%)	63 (83%)	57 (75%)	60 (79%)
PET^b^	Frontal^c^	Temporal		Parietal		PCG/PRC		
Frequency abnormal 5 reads per case (of 71 abnormal cases by majority)	326 (92%)	299 (84%)		323 (91%)		331 (93%)		

*MFL* midfrontal lobe, *STG* superior temporal gyrus, *MTG* middle temporal gyrus, *IPL* inferior parietal lobe, *ACG* anterior cingulate gyrus, *PCG* posterior cingulate gyrus, *PRC* Precuneus, *PVC* primary visual cortex

^a^multiple regions may be simultaneously the region of maximal involvement

^b^BIE assessment also included the subcortical striatal region which was abnormal in 302 assessments (85%)

^c^The frontal lobe was also assessed with the anterior-most aspect of the anterior cingulate in BIE assessment

Analysis of the various histopathological scores against PET assessment showed superiority of plaque based assessments over neuropathological diagnoses. ROC analysis was performed for BIE dichotomy against categorical scores for CERAD (none, sparse, moderate, frequent), NIA-RI AD likelihood (not, low, intermediate high), NIA-AA AD neuropathologic changes (none, low, intermediate, high) and amyloid phase (phase 0–5) and for dichotomised neuropathology (moderate or frequent CERAD, intermediate or high NIA-RI and NIA-AA, and phase 3 or more Thal amyloid phase) against composite cortical SUVRs. In both sets of analyses Thal amyloid phase gave the greatest areas under the curve (>0.95) and this was slightly superior to the ROC analysis of dichotomised BIE against the continuous variable mCERAD_SOT_ of 0.949. (see Additional files 2, 3, 4, 5 and 6).

### The lower limit of β-amyloid detection by PET is of diagnostic relevance

Three observations indicate that the lower limit of detection of plaques by PET is just below the threshold of plaque burden used for the diagnosis of AD, i.e. the lower limit for the detection of plaques by [^18^F]flutemetamol appears to be between the CERAD categories of *sparse* and *moderate* (5-6 neuritic plaques per 100x FoV). Firstly, the probability of an abnormal scan interpretation is almost 0 for the CERAD category of *none*, and almost 1 for the categories of *moderate* and *frequent*, while it is approximately 0.5 for the *sparse* category (Fig. 2a). Secondly, analysis of regional mCERAD_SOT_ values shows that the probability of a region being assessed as abnormal by PET increases between *sparse* and the mid-point between *sparse* (mCERAD_SoT_ =1.0) and *moderate* (mCERAD_SoT_ = 2.0), i.e. at mCERAD_SoT_ = 1.5. Above a score of 1.5, the probability plateaus (Fig. 2b). Thirdly, ROC analysis of mapped regional histometric endpoints (neuritic plaque density, β-amyloid IHC area) and SUVR measures in all 8 neocortical regions for a subset of 32 cases provided optimal sensitivity and specificity in the mCERAD_SOT_ range of 0.9 – 1.1, corresponding to approximately 2-3 neuritic plaques per 100x FoV, in the middle of the *sparse* CERAD category (Additional file 2) and representing β-amyloid levels that are on average lower than those associated with clinicopathologically significant AD (cognitive impairment or dementia due to AD).

### β-amyloid is the primary amyloid target of [^18^F]flutemetamol

Although amyloid in neuritic plaque burden probably accounts for the majority of tracer retention, amyloid (β-pleated sheet) structure is found in several protein aggregates within the brain in AD and other neuropathological degenerative processes. The binding mechanism of Thioflavin-T derivatives (such as flutemetamol) to β-sheets in β-amyloid fibrils is not specific to the primary peptide sequence [6, 41]. These alternative amyloid deposits include other insoluble forms of β-amyloid (diffuse plaques and CAA), as well as other forms of amyloid, such as tau (neurofibrillary tangles [NFT]) and α-synuclein (Lewy bodies). Our subject group contained 2 cases of tangle-predominant dementia and 1 case of Pick’s disease, with minimal co-incidental β-amyloid involvement. All 3 cases were PET negative, suggesting that any cross reactivity to tau at these levels is insufficient to cause ambiguity to interpretation of β-amyloid-based tracer retention. Despite β-amyloid deposits being a commonly observed co-pathology in LBD subjects, only 55% (16/29 cases) were diagnosed with co-incident AD against the NIA-RI criteria, despite 79% (23/29) being PET positive. Most LBD cases without β-amyloid pathology were PET negative and therefore α-synuclein deposits per se may not have a strong contribution to β-amyloid PET imaging, as suggested by previous studies [9, 19, 70]. Of the 13 LBD cases that were not coincident with AD, 7 were mCERAD_SOT_ normal (4 PET negative and 3 PET positive) and 6 were mCERAD_SOT_ abnormal (5/6 PET positive). The 3 PET positive mCERAD_SOT_ normal cases in this disease category represent the only false positive cases in the GE067-026 trial and were scrutinised in greater detail. Two of the three cases (cases 29 and 44) were close to the >1.5 *a priori* threshold for mCERAD_SOT_. As the limit of detection is likely below this threshold (see above), are probably only false positive by virtue of this *a priori* threshold. However, the third case (case 43) had very few neuritic plaques and these were confined to the primary visual cortex, a region not directly assessed in the PET BIE. Two of these 3 cases had not had β-amyloid IHC % area measurements, and so were added to the original 30 cases in a *post hoc* analysis. Both were determined to have a high percentage of tissue area occupied by β-amyloid (Table 1).

Only 3 cases of CAA were recorded in the absence of a diagnosis of AD, and 2 of these were determined to be mCERAD_SOT_ normal. Both were PET negative. For one case CAA involvement was focal and minimal, whereas the other was more extensive, i.e. Vonsattel grade 2, stage 2, and type 1 [56, 64]. With so few cases of CAA in the absence of AD (or accompanying plaque burden), no firm inference can be made regarding the contribution of CAA to tracer retention. Furthermore, composite SUVR_cer_ of AD subjects with and without CAA were comparable (*P* > 0.5, ANOVA). However, indirect evidence that CAA might add to cortical tracer retention may be seen in cases where cortical β-amyloid in the form of neuritic plaques is already close to but below the threshold of detection, i.e. in the 14 amyloid phase 3 cases. In this small subset, CAA was recorded in all 8 cases in which the majority BIE assessment was abnormal and was absent in 5/6 cases where the BIE assessment was normal. The remaining case with majority normal BIE had only focal CAA. The mCERAD_SOT_ scores for these 14 cases were comparable, while the probability of a BIE assessment as abnormal and the cortical composite SUVRs were increased (Fig. 7).

Although the contribution of diffuse plaques to tracer retention is difficult to determine precisely, 2 observations suggest that there is significant binding to diffuse plaques in this study. Firstly, the unequivocally positive PET assessment of case 43 in the virtual absence of neuritic plaques or CAA is possibly explained by the significant cortical diffuse plaque burden, 9.4% of the grey matter stained with β-amyloid IHC (Fig. 6, *Panel c*). Furthermore, any plaque burden in the striatum is predominantly in the form of diffuse plaques [20] and this region was frequently determined to be PET abnormal in our cohort. The term diffuse plaque covers a range of lesions from fleecy amorphous β-amyloid deposits through to plaques that are morphologically better defined but lack the dense core or dystrophic neurites required to be identified as neuritic. Although it has become commonly assumed that diffuse plaques do not contain fibrillar amyloid, electron microscopic observations have indicated that diffuse plaques contain sparse, loosely-textured amyloid fibrils [69] and many diffuse plaques fluoresce weakly with thioflavin S [15] indicating that fibrillar amyloid is often present, although at lower densities than within neuritic or core-only plaques. Also, while the concentration of fibrillar β-amyloid may be substantially lower within diffuse, as opposed to neuritic plaques, the volume occupied by diffuse plaques can be many times greater [43]. These observations support that, while being uncommon occurrence, a heavy diffuse plaque burden in the absence of neuritic plaques may be sufficient to generate an abnormal tracer retention signal.

### Where pathology and PET imaging disagree

In our cohort of 106 subjects, the majority BIE read was disparate from the neuritic plaque assessment in 10 cases, 3 of which were false positives (abnormal PET image with normal pathology) and 7 of which were false negatives (normal PET images with abnormal pathology). Of these 10 cases, half had neuritic plaque density scores near the threshold (see below), but in the other 5 cases (cases 27, 28, 41, 42 and 43) there was no overt reason for the disparity, as the neuritic plaque density score was not considered borderline. Only one of the unexplained cases (case 43) was a false positive without a significant neuritic plaque burden (mCERAD_SOT_ = 0.3, see earlier comments on heavy diffuse plaque burden in this case). A CERAD assessment of *moderate* entered onto the case report form was not substantiated by the mCERAD_SOT_ score nor the neuropathology report, and was deemed a transcription error from “moderate diffuse plaque frequency,” which was recorded in the report. However, whilst there is little doubt that the cortical plaque burden was low, the % grey-matter area stained with β-amyloid IHC was high (see Fig. 3), the amyloid phase was 4, the Braak stage was III, and the patient had a history of dementia. Because of the scarcity of neuritic plaques and presence of significant Lewy bodies, the primary diagnosis for this case was LBD. Interestingly, this case would be considered as having intermediate AD neuropathology changes if using the more recent National Institute on Aging-Alzheimer's Association (NIA-AA) diagnostic criteria [25].

The remaining 4 cases were false negatives with unequivocally abnormal β-amyloid burdens. The mCERAD_SOT_ scores for these cases were 1.8, 2.1, 1.9, and 2.7 for cases 27, 28, 41, and 42, respectively (Table 1). It is possible that the increased partial volume effect produced by severe neocortical atrophy may be at least partially responsible for the misclassification, although equivalent atrophy was seen in many patients whose images were correctly interpreted. Three of these 4 cases had a modest neuritic plaque burden and only one of them had a heavy neuritic plaque burden (mCERAD_SOT_ = 2.7, case 42). This last case should not have escaped PET detection. Indeed, in this case (case 42), 2/5 readers recorded abnormal retention in 4 of the 5 regions assessed, and all 5 readers reported low confidence in interpreting this image.

Most disparities occurred in cases with *sparse* or *moderate* neuritic plaque densities. The differentiation between *sparse* (1 to 5 plaques) and *moderate* (6 to 19 plaques) may differ by one plaque per field of view, and some misclassification is therefore inevitable when the underlying pathology is close to a threshold. Indeed, half of the disparity cases had β-amyloid pathology categories straddling the threshold for dichotomy (*sparse* and *moderate*) and amyloid phase 3 (4/10). Of the 7 false negative cases, only 1/7 (14%) was abnormal for all 8 cortical regions assessed compared to 39/69 (56%) in true positive cases. The remaining cases varied from 1 to 5 regions abnormal (out of the 8 sampled). Furthermore, of 6 cases classified as abnormal on the basis of abnormal pathology in a single region, half were classified as false negatives. However, it is important to determine whether additional factors could contribute to misclassification.

In a *post-hoc* analysis, we classified cases as equivocal by either PET or pathology against the following defined criteria; *equivocal by pathology* if the mCERAD_SOT_ max was 1.25 – 1.75, based on a theoretical influence of 1 aberrant measure on a regional assessment; 5 slides scored as 1.5 and one slide scored as 0 (mCERAD_SOT_ = 1.25) or 3 (mCERAD_SOT_ = 1.75); *equivocal by PET* if fewer than 4/5 readers concurred; i.e., if majority assessment was 3:2, or if reader confidence was low (mean confidence of less than 4 on a scale of 1–5). A total of 24 cases were equivocal by either PET or pathology criteria; 6 cases were equivocal by both criteria. Fourteen cases were considered as equivocal by pathology, 12 with borderline mCERAD_SOT_ and 6 with mCERAD_SOT_ > 1.5 based on a single region (4 cases met both criteria). Sixteen cases were equivocal by PET, all with low reader confidence and 4 of these also by split majority (3:2 reader ratio). Interestingly, of these 16 cases, half were amyloid phase 3, or the preclinical to clinical transition phase (*P* < 0.01, *χ* ^2^ test; observed vs. expected), indicating that at this intermediate level of AD β-amyloid pathology, the images are less easy to interpret. Indeed, of the 14 phase 3 cases, only half were interpreted as positive by BIE majority. Of the 10 cases that were false positive or false negative by PET majority read, half were equivocal by pathology.

## Discussion

The data presented in this study examined the histopathology underlying [^18^F]flutemetamol tracer retention and PET image interpretation. The cohort of 106 end-of-life subjects represented a broad and continuous spectrum of β-amyloid pathology and other comorbidities, including several cases where the neuritic plaque burden was diagnostically equivocal. Such cases are valuable for a critical assessment of imaging agent performance and diagnostic limitations.

No subjects were designated as abnormal by majority PET interpretation in the absence of fibrillar β-amyloid plaques. This study, and others using alternative β-amyloid PET tracers, demonstrate that neocortical neuritic plaque burden is the most important β-amyloid histopathological feature underlying tracer retention [11]. Key to demonstrating this was the reliable assessment of the neuritic plaque burden through multiple regional assessments that considerably improved upon a single point CERAD assessment, which is vulnerable to sampling bias and inter-reader variability [1]. Additionally, rather than limiting histopathological or PET analysis to the 3 anatomic regions originally recommended by CERAD, global cerebral tracer retention, which is reflective of global cerebral β-amyloid deposition, is validated by the good efficacy of multiple trials and tracers [11, 12, 28, 31, 35–38, 47, 50–52, 65–67]. Indeed, interpretation of PET images may be enhanced by the inclusion of medial cerebral gyri, notably the cingulate and precuneus, regions that have not previously formed part of the standard neuropathology assessment. In practice, assessment as abnormal based upon a single region is unusual. Only a single case (<1%) in our cohort was designated as abnormal based upon a single positive PET region. Interestingly this case has borderline amyloid burden (case 49). Although tissue sections are relatively thin, in coronal cross section they cover a cortical area (3–5 cm) comparable to that assessed by PET and are unlikely to be a cause of discrepancy between the pathology and PET, especially if 8 (or more) cortical regions are assessed and multiple plaque measures per section also mitigated plaque heterogeneity. Furthermore, there is likely to be merit in assessment of subcortical areas such as the striatum [5] which is significant in amyloid progression [25, 57].

Unsurprisingly, the single most confounding factor in image interpretation is equivocal pathology, where the underlying histopathology is near the limit of reliable detection in PET images. In the present multi-reader study, this was manifest in low reader confidence and disagreement between the multiple readers. In clinical practice, it will probably be beneficial to consider a categorical assessment of “equivocal”, an option which was not made available to readers during efficacy trials due to the regulatory requirement of dichotomy. Importantly, the detectable neuritic plaque burden by [^18^F]flutemetamol imaging is diagnostically relevant in that it aligns with what is the accepted distinction between normal and pathologic levels of β-amyloid burden used for AD diagnosis. However, the probability of a positive PET assessment is non-zero in the sparse CERAD category and over a range of mCERAD_SOT_ scores between 1 and 2 (sparse to moderate). It is possible that varying PET methodology may be able to alter the threshold for calling a scan positive. Paradoxically, better PET sensitivity will cause worse accuracy for predicting cognitive impairment due to AD as too many sparse-plaque cases will be called PET positive. On the other hand, detecting more of the sparse plaque cases may be better for subject selection for prevention trials for AD, as the chance of success for such trials will presumably be better for those subjects at an earlier disease stage. The lower limit of detection of plaques by amyloid tracers may vary by anatomical region and partial volume effects, but it appears likely from these results that flutemetamol may detect cortical plaques below the threshold normally associated with a clinically significant “moderate” plaque burden and further quantitative methodological studies are needed.

Mixed pathologies in this cohort enabled us to investigate how co-incident pathology may impact PET image interpretation. Amyloidopathies other than those containing β-amyloid (i.e. tauopathies and synucleinopathies) did not clearly interfere with β-amyloid PET interpretation, most likely through a lack of detectable signal. We cannot comment on the potential for a weak signal in areas not assessed in this study, e.g. mesial temporal for Tau). However, other forms of β-amyloid deposits such as diffuse plaques and CAA possibly contribute to overall tracer retention. These forms of β-amyloid are often associated with AD, but are also seen as separate entities, in that they are occasionally recorded in the absence of typical AD neuritic plaque and NFT neuropathology. The β-amyloid burden in the form of diffuse plaques is variable, as the term covers a range of lesions with varying β-amyloid density and fibrillar β-amyloid content. Nonetheless, frequent diffuse plaques alone were sufficient for PET positivity in the cortex of one case (case 43) and in the striatum in 9 cases - in agreement with in vitro studies of flutemetamol and the chemically related PiB, which lightly stain diffuse plaques [28].

While at first impression, the inability to discriminate between a significant diffuse plaque burden and a more modest neuritic plaque burden may appear to be a disadvantage, this may not be a critical weakness, as cases with a heavy load of cortical diffuse plaques but absent or scarce neuritic plaques are uncommon. Also, more importantly, such cases may meet current diagnostic criteria for clinicopathologically significant AD as exemplified by case 43, which met the 2012 AD criteria [25]. Indeed, such tracer retention by diffuse plaques may be a diagnostic advantage in regions such as the striatum, where plaques are predominantly diffuse [16] yet are diagnostically indicative of an advanced β-amyloid phase [58], and may also be used to predict associated NFT stage [16]. In this latter study, striatal β-amyloid identified AD pathology in the presence of Lewy body disease, which is analogous to some of our subjects. Striatal β-amyloid detection may be particularly important in some cases of presenilin-1 (PS1)-associated AD where the striatum may be the first area affected [34]. However, to our knowledge, we did not have any PS1 mutations in this study. The presence of plaques in the striatum represents a transition from amyloid phase 2 (Normal) to phase 3 (Abnormal) [58]. However, in the early stages of phase 3 amyloid burden is likely to be insufficient to be detected by PET imaging and the burden in the striatum may not become sufficient until phase 4. Indeed, in the 6 cases where PET assessment was positive in the cortex but negative in the striatum 4 were classified as phase 3 and 2 were classified as phase 4. We are currently assessing the significance of striatal imaging in further depth (see also changes to the AD diagnostic criteria below).

While the β-amyloid in cortical diffuse plaques may not often be clinically significant on its own, the deposits of β-amyloid in CAA confer a substantial risk of lobar haemorrhage. Therefore, the ability of β-amyloid tracers to identify CAA is of considerable interest [3, 30]. While others have reported positive PET signal with CAA using the chemically related [^11^C]PiB [3, 30], our cohort contained no cases with CAA in the absence of at least some fibrillar β-amyloid plaques. Nonetheless, CAA may contribute to cerebral PET positivity in a subset of cases supporting reports that CAA is detectable by others [2] but that, although substantial amounts of β-amyloid may be present in vasculature, tracer retention by CAA is modest compared to that by cortical neuritic plaques. Our analysis did not include PET image assessment of the occipital lobe, where others report predominant CAA tracer retention [2]. Any potential clinical utility of β-amyloid tracers with regards to CAA may rest mainly in the ability to rule out CAA with a negative scan, in much the same way as a negative scan also rules out AD.

Significant cortical atrophy in end-stage Alzheimer’s disease can complicate the interpretation of [^18^F]flutemetamol PET images, which requires assessment of pathological gray-matter retention adjacent to normal white-matter retention pattern. In most circumstances, this is relatively straightforward, but may be complicated by significant atrophy of the cortical ribbon, rendering differentiation between grey and white matter more difficult in the PET only image. As atrophy is a common feature of advanced AD, atrophy in the cohort as a whole was generally more frequent with increased positive PET images, reflecting the underlying advanced AD. However, in cases where atrophy was recorded as severe, the probability of a positive PET interpretation decreased without a concurrent drop in reader confidence. Four of the seven false negative cases were noted to have severe atrophy during gross examination at post-mortem. It therefore appears likely that while equivocal pathology is associated with low reader confidence - and likely accounts for some of the false negative cases - atrophy probably accounts for some of the false-negative interpretations without a concurrent drop in reader confidence. While amyloid pathology is generally symmetric in distribution, focal atrophy present in some of our cases showed distinct laterality, resulting in an asymmetric PET image. The electronic training program for image interpretation [54, 63] provides guidance on how to interpret images with such focal atrophy.

Our results demonstrate an association of [^18^F]flutemetamol PET with β-amyloid deposition in the neocortex, while PET positivity correlated less well with a neuropathologically-supported diagnosis of AD. The AD diagnostic criteria used here were the National Institute of Aging – Reagan Institute criteria [26]. The association between neuritic β-amyloid plaques and NFT is imperfect [21], and so any measure of β-amyloid in the cortex cannot ‘rule in’ AD, as it is unable to measure NFT. Instead, its utility is primarily intended to ‘rule out’ AD if the PET scan is negative. However, the PET detection of striatal plaque burden may offer a more sensitive assessment of AD progression [16] and a recent revision of AD diagnostic guidelines [25], which includes β-amyloid phase analysis [58], indicates that striatal imaging may refine AD assessment with β-amyloid tracers.

The GE067-026 trial findings presented here for [^18^F]flutemetamol are consistent with those reported previously in cerebral biopsy trials and by other β-amyloid imaging agents in end-of-life subjects, such as the chemically related [^11^C]PiB and chemically less similar [^18^F]AV-45, supporting the utility of β-amyloid imaging in vivo [11, 28, 67]. The ‘holy grail’ for subject selection for prevention trials would be detection of early or preclinical plaque burden with a detection limit clearly below the levels that are associated with significant cognitive decline. The probability of a positive PET image interpretation in this study was 50% in the sparse category, suggesting that this may be possible, but it seems likely that analysis of other regions, such as the striatum or, better yet, a measurable, reliable quantification method, would likely improve the lower detection limit, enabling assessment of β-amyloid in preclinical AD, a stage of disease almost certainly more amenable to preventative treatment strategies or disease-modifying therapeutics. In this context, it is necessary to note that recent studies reported that amyloid PET only detects cases with already advanced amyloid phases (3 and higher) whereas initial phases (1 and 2) could not be identified with [^18^F]flutemetamol or [^11^C]PIB [45, 55]. Conversely, increasing the sensitivity of PET β-amyloid scans may decrease the specificity to predict clinically significant AD neuropathology. It is possible that PET image interpretation methods might be adjustable to fit the clinical setting, with a less sensitive scan (similar to those currently in use) being used in the setting of dementia and a more sensitive scan used in the setting of selection of cognitively normal, β-amyloid positive subjects for AD prevention trials.

β-amyloid PET imaging studies with neuropathology validation in post-mortem brains [2, 9–11, 27, 28, 32, 33, 45, 53, 62] or biopsy brain tissues [37, 50, 51, 67] are usually limited by small numbers of subjects and could be biased by variable time between PET acquisition and autopsy or biopsy. However, our study of 106 subjects is a relatively large cohort and the influence of variable PET to autopsy interval was not a significant factor [49]. Small numbers of disease transition subsets, such as 14 amyloid phase 3 subjects, show interesting results – low reader confidence, low inter-reader agreement and possible influence of CAA – and while analyses reach statistical significance, conclusions drawn from small numbers such as these require further research to substantiate or refute these findings.

## Conclusions

In summary, examination of data from an end-of-life clinical trial of Flutemetamol F 18 Injection demonstrated a high specificity and sensitivity for β-amyloid pathology, and detection of a diagnostically relevant neuritic plaque burden. Abnormal PET scans were always associated with underlying AD histopathology and equivocal PET scan reads usually indicated some underlying AD histopathology, possibly at levels usually associated with cognitive normality or early mild cognitive impairment. Other co-morbidities did not interfere with image interpretation, although advanced cortical atrophy could hamper interpretation even in advanced AD cases. For the future, opportunities to quantify or automate the interpretation of PET images [59] and the study of greater numbers of subjects in the intended target population may further refine the clinical utility of β-amyloid PET imaging following Flutemetamol F 18 Injection.

## Additional files

Additional file 1: Example of the recording of pathology sampling to enable mapped cortical tracer retention SUVRs to be measured for correlation. Regions sampled from the lateral surface include the Midfrontal lobe (MFL), Superior Temporal Gyrus (STG), Middle Temporal Gyrus (MTG) and Inferior Parietal Lobe. Regions sampled from the medial surface include Anterior Cingulate Gyrus (ACG), Posterior Cingulate Gyrus (PCG), Precuneus (PRC) and Primary Visual Cortex (PVC). (DOC 90 kb)
Additional file 2: Receiver operator characteristic (ROC) analysis of regional histometric measures and standard retention value ratios. Regional analysis of 32 brains provided retention correlation with plaque burden and provided a large data set (*N* = 256) with which to estimate the lower limit of detection. For regional analysis SUVR baselines which varied between regions due to variable adjacent white-matter signal bleed were used to define a threshold for each region. Thresholds were determined post-hoc by ROC analysis as the maximal sum of the sensitivity (true positive rate) and specificity (1 - false-positive rate). (DOC 59 kb)
Additional file 3: ROC Analysis - Dichotomised BIE (majority read) vs categorical neuropathology. Comparison of the 4 histopathological criteria of assigning abnormal amyloid against a dichotomised (Abnormal/Normal) majority blinded PET Image evaluation. The 4 criteria shown are CERAD [43], National Institutes of Ageing – Reagan Institute AD diagnosis [26], National Institutes of Ageing – Alzheimer’s Association AD diagnosis [25] and Amyloid phase (Phase 3 or greater) [58]. (DOC 201 kb)
Additional file 4: ROC Analysis: Dichotomised pathology vs continuous SUVR. Comparison of 5 histopathological criteria of assigning abnormal amyloid against PET signal quantification (SUVR). Histopathological criteria used were: CERAD [43] moderate or frequent = abnormal; National Institutes of Ageing – Reagan Institute [26] Intermediate or High = abnormal; National Institutes of Ageing – Alzheimer’s Association [25] Intermediate or High = abnormal; Thal 3+ Thal amyloid phase [58] of 3 or higher = abnormal; Thal 4+ Thal amyloid phase [58] of 4 or 5 = abnormal. (DOC 241 kb)
Additional file 5: Clinical Status of subjects at PET Scan. Spontaneously recorded clinical notes captured at the time of PET scan. The PET category provided reflects PET majority image assessment against pathology dichotomy normal/abnormal by mCERADSOT. (DOC 155 kb)
Additional file 6: Amyloid plaque frequencies. In addition to the neuritic plaque frequencies assessed on Bielschowsky silver stained sections, semiquantitative plaque frequencies of none, sparse, moderate or frequent [43] were obtained from β-amyloid stained sections. In addition to the frequencies recorded in the CERAD neocortical regions, the Amyloid phase was recorded after examining multiple cortical, subcortical, midbrain and hindbrain regions [58]. (DOC 112 kb)
