# Supplementary material for: Post-mortem histopathology underlying β-amyloid PET imaging following flutemetamol F 18 injection
Source: Acta Neuropathol Commun. 2016 Dec 12;4:130. doi: 10.1186/s40478-016-0399-z (PMC5154022; doi:10.1186/s40478-016-0399-z)
Supplement: Additional file 1: — Example of the recording of pathology sampling to enable mapped cortical tracer retention SUVRs to be measured for correlation. Regions sampled from the lateral surface include the Midfrontal lobe (MFL), Superior Temporal Gyrus (STG), Middle Temporal Gyrus (MTG) and Inferior Parietal Lobe. Regions sampled from the medial surface include Anterior Cingulate Gyrus (ACG), Posterior Cingulate Gyrus (PCG), Precuneus (PRC) and Primary Visual Cortex (PVC). (DOC 90 kb) [file 40478_2016_399_MOESM1_ESM.doc]

**Additional file 1 Example of the recording of pathology sampling to enable mapped cortical tracer retention SUVRs to be measured for correlation.**


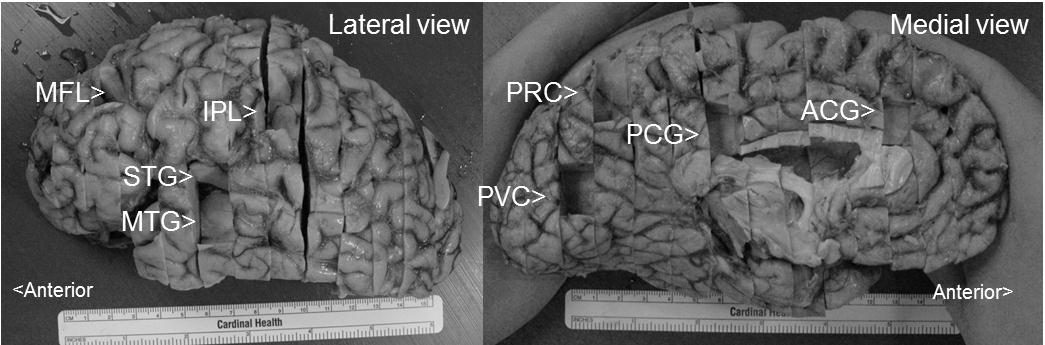


*MFL* midfrontal, *STG* superior temporal gyrus, *MTG* middle temporal gyrus, *IPL* inferior parietal lobe, *ACG* anterior cingulate gyrus, *PCG* posterior cingulate gyrus, *PRC* Precuneus, *PVC* primary visual cortex
