# Supplementary material for: Post-mortem histopathology underlying β-amyloid PET imaging following flutemetamol F 18 injection
Source: Acta Neuropathol Commun. 2016 Dec 12;4:130. doi: 10.1186/s40478-016-0399-z (PMC5154022; doi:10.1186/s40478-016-0399-z)

**Additional file 1**

Receiver operator characteristic (ROC) analysis of regional histometric measures and standard retention value ratios

Regional analysis of 32 brains provided retention correlation with plaque burden and provided a large data set (N = 256) with which to estimate the lower limit of detection. For regional analysis SUVR baselines which varied between regions due to variable adjacent white-matter signal bleed were used to define a threshold for each region. Thresholds were determined *post-hoc* by ROC analysis as the maximal sum of the sensitivity (true positive rate) and specificity (1 - false-positive rate). Using the ROC-analysis PET thresholds, the optimum BSS score was then determined to estimate the lower limit of neuritic plaques detectable by [^18^F]flutemetamol PET. For the eight regions sampled, the regional mCERAD_SOT_ score for the lower limit ranged from 0.4 to1.3 and overall was 0.9-1.1 for ROC analysis set SUVR thresholds. Modelling the neuritic plaque density corresponding to a given mCERAD_SOT_ value used the best fit equation;

mCERAD_SOT_ = 0.6428 x (neuritic plaque count / 100x field of view)^0.4585^ or

Neuritic plaque count ~ 2.07 x mCERAD_SOT_^2.18^ plaques per 100x FoV.

Therefore, a regional mCERAD_SOT_ of 1.1 corresponds to approximately 2.5 plaques per 100x field of view or approximately half of the diagnostically relevant *a priori* threshold of 1.5 representing the boundary between the CERAD categories of sparse and moderate.

*A priori* ROC analysis fitted curve Maximum likelihood estimation of a binormal ROC curve from continuously distributed test results

Java translation by John Eng, M.D. The Russell H. Morgan Department of Radiology and Radiological Science Johns Hopkins University, Baltimore, Maryland, USA Version 1.0.1, Aug 2006 Trapezoidal (Wilcoxon) AUC = 0.9355


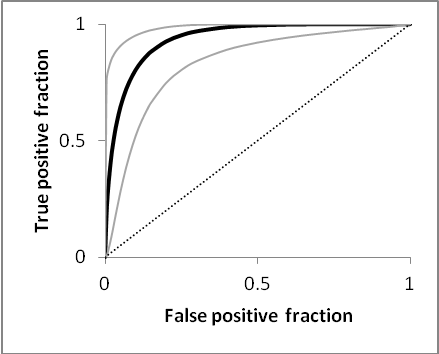

Supplement: Additional file 2: — Receiver operator characteristic (ROC) analysis of regional histometric measures and standard retention value ratios. Regional analysis of 32 brains provided retention correlation with plaque burden and provided a large data set (N = 256) with which to estimate the lower limit of detection. For regional analysis SUVR baselines which varied between regions due to variable adjacent white-matter signal bleed were used to define a threshold for each region. Thresholds were determined post-hoc by ROC analysis as the maximal sum of the sensitivity (true positive rate) and specificity (1 - false-positive rate). (DOC 59 kb) [file 40478_2016_399_MOESM2_ESM.doc]
