# Supplementary material for: Post-mortem histopathology underlying β-amyloid PET imaging following flutemetamol F 18 injection
Source: Acta Neuropathol Commun. 2016 Dec 12;4:130. doi: 10.1186/s40478-016-0399-z (PMC5154022; doi:10.1186/s40478-016-0399-z)
Supplement: Additional file 3: — ROC Analysis - Dichotomised BIE (majority read) vs categorical neuropathology. Comparison of the 4 histopathological criteria of assigning abnormal amyloid against a dichotomised (Abnormal/Normal) majority blinded PET Image evaluation. The 4 criteria shown are CERAD [43], National Institutes of Ageing – Reagan Institute AD diagnosis [26], National Institutes of Ageing – Alzheimer’s Association AD diagnosis [25] and Amyloid phase (Phase 3 or greater) [58]. (DOC 201 kb) [file 40478_2016_399_MOESM3_ESM.doc]

| **Dichotomised BIE (majority read) vs categorical neuropathology** | | | |
| --- | --- | --- | --- |
| **CERAD** | **NIA-RI** | **NIA-AA** | **Thal** |
|  |  |  |  |
| Number of Cases: 106 | Number of Cases: 106 | Number of Cases: 106 | Number of Cases: 106 |
| Number Correct: 91 | Number Correct: 88 | Number Correct: 91 | Number Correct: 94 |
| Accuracy: 85.8% | Accuracy: 83.0% | Accuracy: 85.8% | Accuracy: 88.7% |
| Sensitivity: 87.5% | Sensitivity: 79.2% | Sensitivity: 87.5% | Sensitivity: 100.0% |
| Specificity: 82.4% | Specificity: 91.2% | Specificity: 82.4% | Specificity: 64.7% |
| Pos Cases Missed: 9 | Pos Cases Missed: 15 | Pos Cases Missed: 9 | Pos Cases Missed: 0 |
| Neg Cases Missed: 6 | Neg Cases Missed: 3 | Neg Cases Missed: 6 | Neg Cases Missed: 12 |
| (A rating of moderate or greater is considered positive.) | (A rating of intermediate or greater is considered positive.) | (A rating of intermediate or greater is considered positive.) | (A rating of phase 3 or greater is considered positive.) |
| Fitted ROC Area: 0.93 | Fitted ROC Area: 0.94 | Fitted ROC Area: 0.927 | Fitted ROC Area: 0.966 |
| Empiric ROC Area: 0.912 | Empiric ROC Area: 0.889 | Empiric ROC Area: 0.892 | Empiric ROC Area: 0.956 |
| 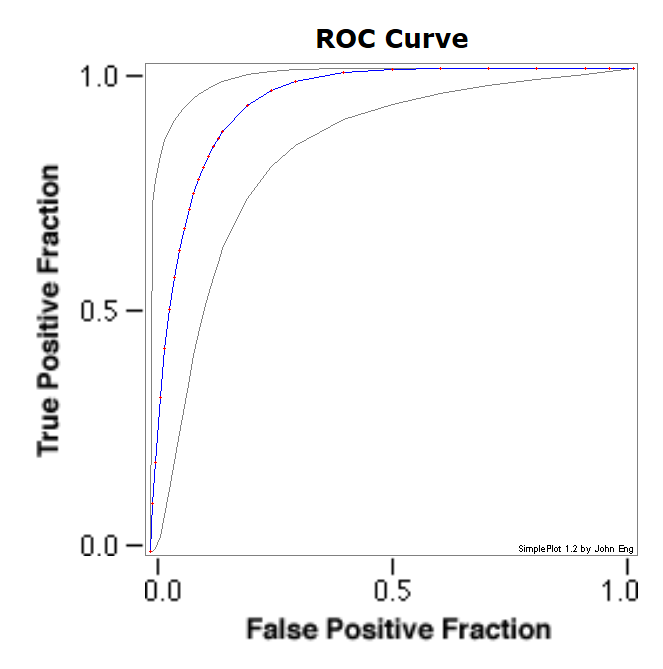 | 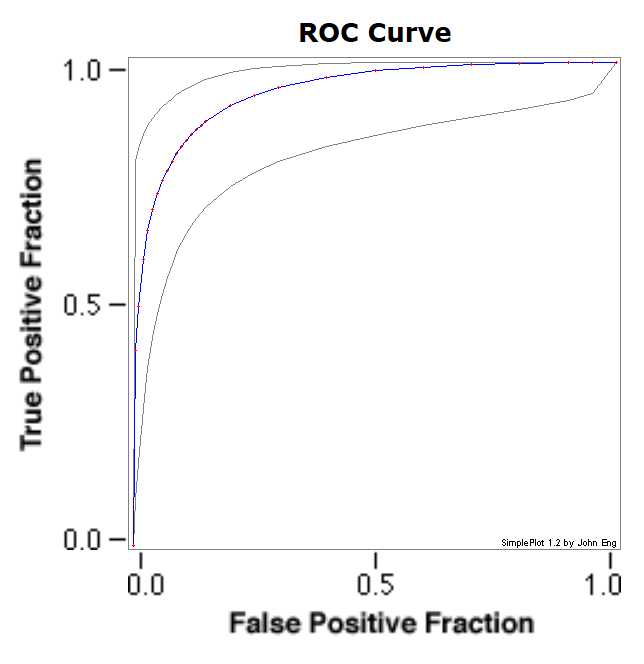 | 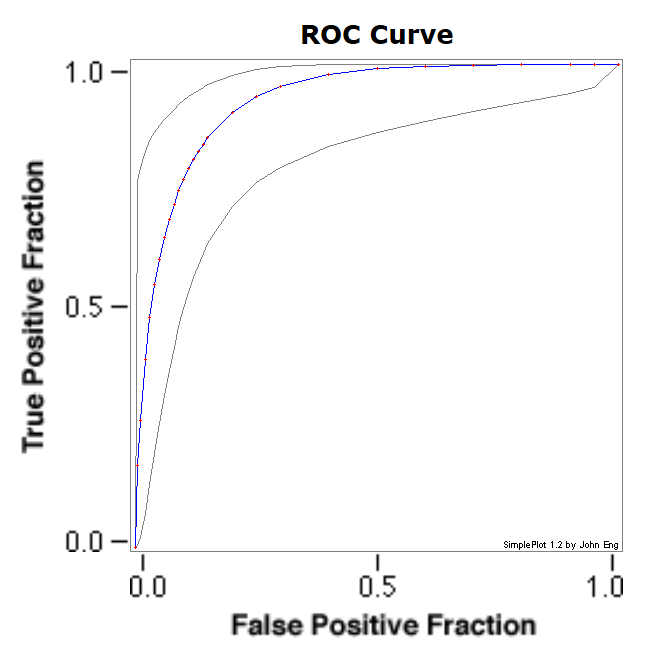 | .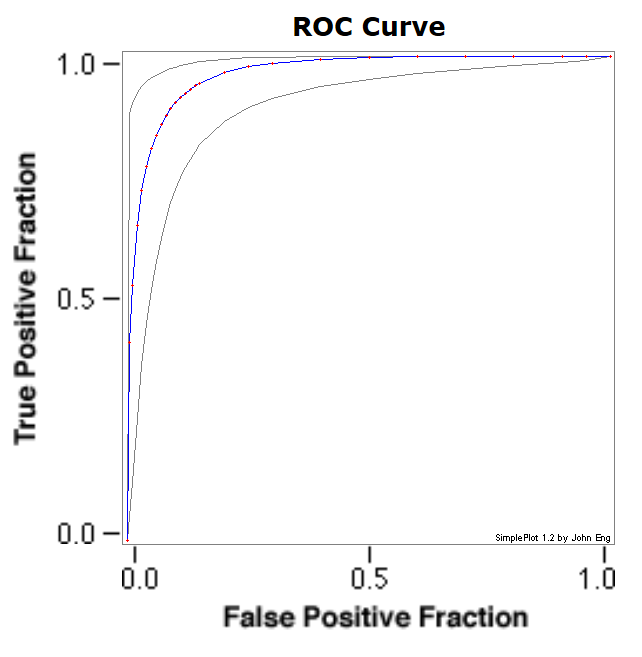 |

BIE majority read; 3/5, 4/5 or 5/5 readers abnormal = abnormal.
