# Supplementary material for: Post-mortem histopathology underlying β-amyloid PET imaging following flutemetamol F 18 injection
Source: Acta Neuropathol Commun. 2016 Dec 12;4:130. doi: 10.1186/s40478-016-0399-z (PMC5154022; doi:10.1186/s40478-016-0399-z)
Supplement: Additional file 4: — ROC Analysis: Dichotomised pathology vs continuous SUVR. Comparison of 5 histopathological criteria of assigning abnormal amyloid against PET signal quantification (SUVR). Histopathological criteria used were: CERAD [43] moderate or frequent = abnormal; National Institutes of Ageing – Reagan Institute [26] Intermediate or High = abnormal; National Institutes of Ageing – Alzheimer’s Association [25] Intermediate or High = abnormal; Thal 3+ Thal amyloid phase [58] of 3 or higher = abnormal; Thal 4+ Thal amyloid phase [58] of 4 or 5 = abnormal. (DOC 241 kb) [file 40478_2016_399_MOESM4_ESM.doc]

| **Dichotomised pathology vs continuous SUVR (composite cer of BIE regions)** | | | | |
| --- | --- | --- | --- | --- |
| **CERAD** | **NIA-RI** | **NIA-AA** | **Thal 3+** | **Thal 4+** |
|  |  |  |  |  |
| Total Cases: 106 | Total Cases: 106 | Total Cases: 106 | Total Cases: 106 | Total Cases: 106 |
| Positive Cases: 69 | Positive Cases: 60 | Positive Cases: 69 | Positive Cases: 84 | Positive Cases: 70 |
| Negative Cases: 37 | Negative Cases: 46 | Negative Cases: 37 | Negative Cases: 22 | Negative Cases: 36 |
|  |  |  |  |  |
| Fitted ROC Area: 0.813 | Fitted ROC Area: 0.833 | Fitted ROC Area: 0.89 | Fitted ROC Area: 0.931 | Fitted ROC Area: 0.952 |
| 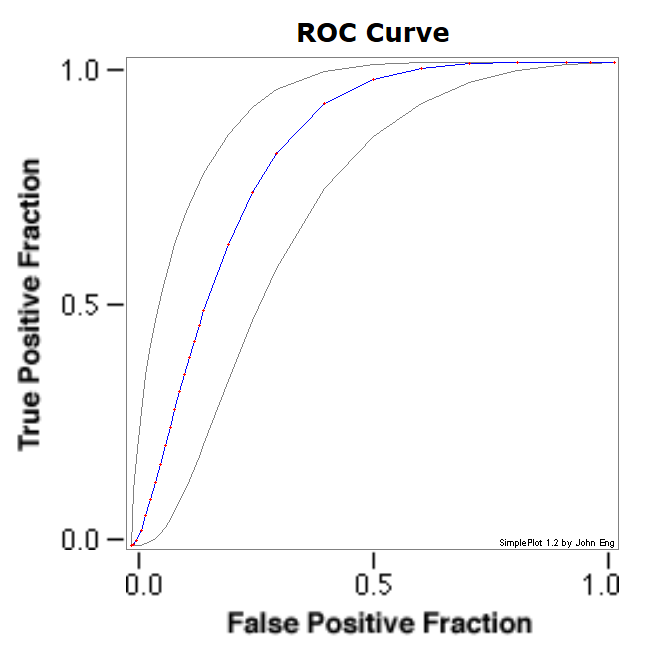 | 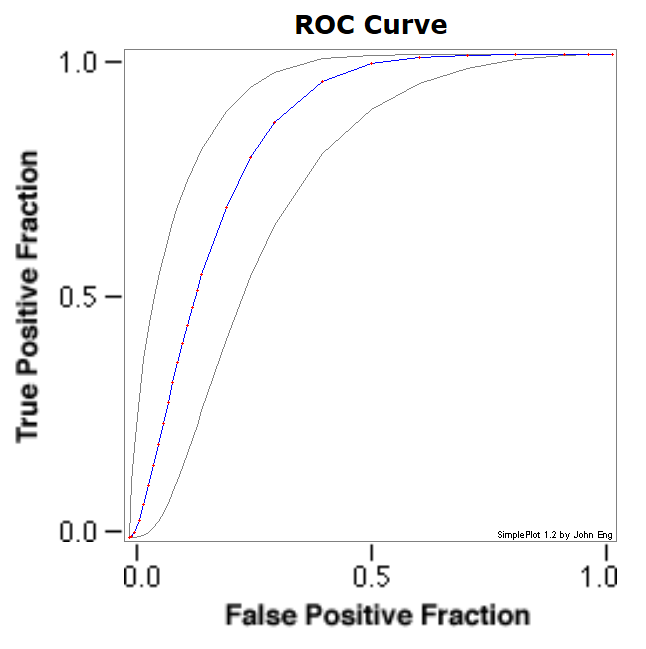 | 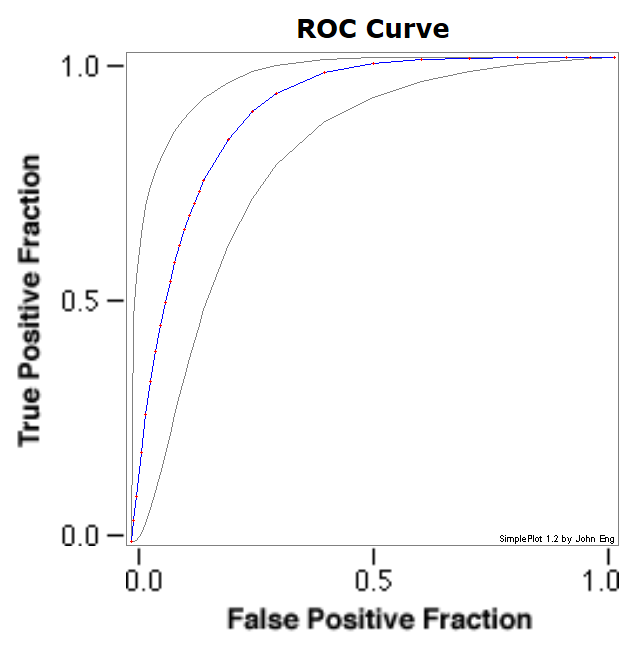 | 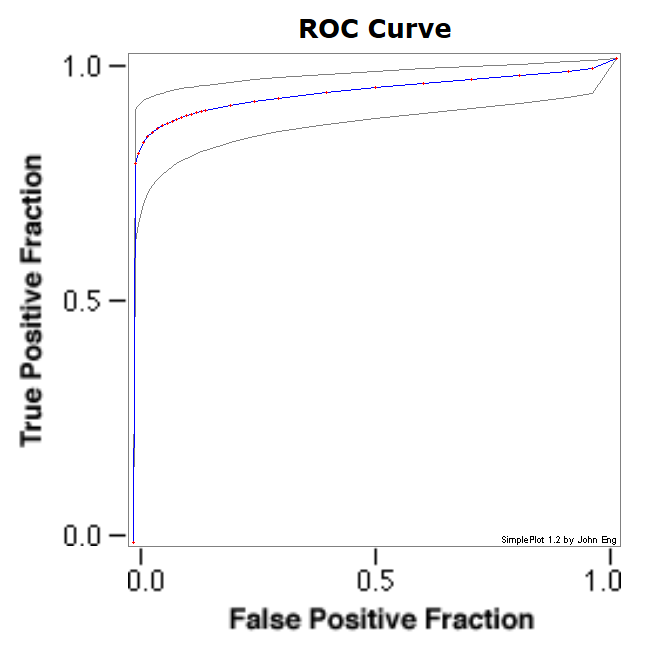 | 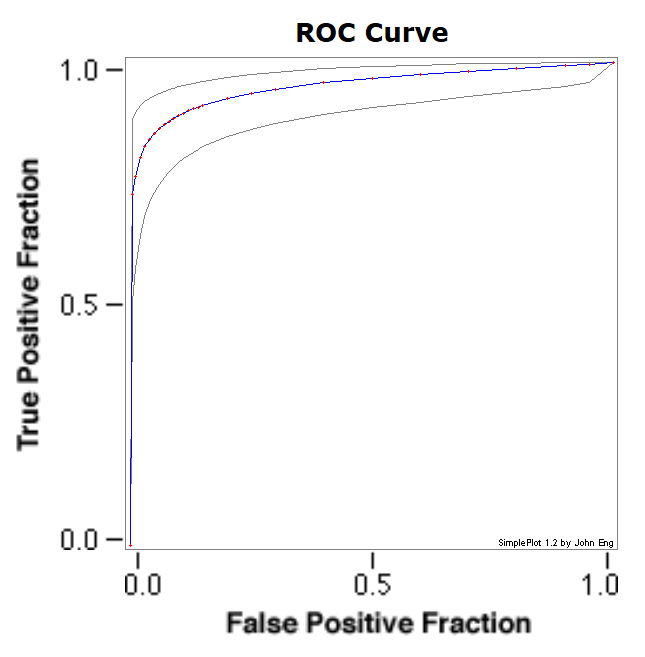 |

Dichotomy: CERAD moderate or frequent = abnormal; NIA-RI Intermediate or High = abnormal; NIA-AA Intermediate or High = abnormal; Thal 3+ Thal amyloid phase 3 or higher = abnormal; Thal 4+ Thal amyloid phase 4 or 5 = abnormal
