# Supplementary material for: Post-mortem histopathology underlying β-amyloid PET imaging following flutemetamol F 18 injection
Source: Acta Neuropathol Commun. 2016 Dec 12;4:130. doi: 10.1186/s40478-016-0399-z (PMC5154022; doi:10.1186/s40478-016-0399-z)
Supplement: Additional file 5: — Clinical Status of subjects at PET Scan. Spontaneously recorded clinical notes captured at the time of PET scan. The PET category provided reflects PET majority image assessment against pathology dichotomy normal/abnormal by mCERADSOT. (DOC 155 kb) [file 40478_2016_399_MOESM5_ESM.doc]

| **Clinical Status of subjects at PET Scan** | | | | | | |
| --- | --- | --- | --- | --- | --- | --- |
| **Case number** | **PET category^a^** | **Sex** | **Weight**  **(kg)** | **Time PET to**  **Death (days)** | **Weight Category^b^** | **Clinical Notes (General condition)^c^** |
| 1 | True Negative | F | 65 | 360 | Overweight | - |
| 2 | True Negative | M | 63.6 | 17 | Normal | Cachectic, pale |
| 3 | True Negative | M | 61.8 | 568 | Normal | - |
| 4 | True Negative | M | 60 | 130 | Normal | - |
| 5 | True Negative | M | 72.7 | 433 | Normal | - |
| 6 | True Negative | F | 55.9 | 145 | Normal | - |
| 7 | True Negative | M | 80 | 16 | Normal | Lethargic |
| 8 | True Negative | M | 66.8 | 32 | Normal | - |
| 9 | True Negative | M | 109.1 | 131 | Obese Class I | Frail elderly in wheelchair NCS |
| 10 | True Negative | F | 95.5 | 34 | Obese Class II | - |
| 11 | True Negative | F | 84.5 | 393 | Obese Class I | - |
| 12 | True Negative | M | 60.5 | 374 | Normal | - |
| 13 | True Negative | M | 57.3 | 170 | Normal | Decrease weight, decrease muscle mass |
| 14 | True Negative | M | 72.3 | 155 | Normal | - |
| 15 | True Negative | F | 81.8 | 10 | Obese Class I | - |
| 16 | True Negative | M | 76.8 | 12 | Overweight | - |
| 17 | True Negative | F | 45.5 | 105 | Underweight | Decrease muscle mass lower extremities |
| 18 | True Negative | F | 54.5 | 115 | Overweight | Lethargic, somnolent, minimally responsive |
| 19 | True Negative | F | 44.5 | 78 | Normal | - |
| 20 | True Negative | F | 50.9 | 24 | Normal | Bedridden condition |
| 21 | True Negative | F | 42.7 | 210 | Underweight | - |
| 22 | True Negative | F | 45.5 | 69 | Normal | - |
| 23 | True Negative | M | 81.8 | 142 | Normal | - |
| 24 | True Negative | F | 35.5 | 76 | Underweight | Bed ridden, decrease muscle mass |
| 25 | True Negative | F | 52.3 | 137 | Normal | Chronically ill (NCS) |
| 26 | **False Negative** | M | 55.1 | 11 | Underweight | - |
| 27 | **False Negative** | M | 55.9 | 189 | Normal | Thin |
| 28 | **False Negative** | F | 50.9 | 212 | Normal | - |
| 29 | **False Positive** | F | 62.3 | 131 | Overweight | - |
| 30 | True Positive | F | 45.4 | 630 | Normal | - |
| 31 | True Positive | F | 26.8 | 132 | Very severely underweight | Cachectic emaciated (NCS) |
| 32 | True Positive | F | 56.8 | 311 | Normal | Right knee scar |
| 33 | True Positive | F | 41.8 | 118 | Underweight | - |
| 34 | True Positive | M | 59.1 | 2 | Normal | Staring, open-mouthed. Mute, myoclonus |
| 35 | True Positive | F | 41.8 | 19 | Normal | - |
| 36 | True Positive | F | 50 | 329 | Underweight | - |
| 37 | True Positive | F | 57.1 | 550 | Normal | - |
| 38 | True Negative | M | 43.6 | 19 | Severely underweight | Failure to thrive, appearance no dentition |
| 39 | True Negative | M | 63.6 | 64 | Normal | Decreased mobility |
| 40 | **False Negative** | M | 69.1 | 349 | Normal | Left forehead with status post burr hole (very old) |
| 41 | **False Negative** | M | 60 | 323 | Normal | - |
| 42 | **False Negative** | M | 102.7 | 22 | Obese Class I | - |
| 43 | **False Positive** | F | 45.5 | 193 | Normal | - |
| 44 | **False Positive** | M | 58.9 | 84 | Normal | - |
| 45 | True Positive | M | nd | 373 |  | - |
| 46 | True Positive | F | 51.8 | 127 | Normal | - |
| 47 | True Positive | M | 78.6 | 395 | Overweight | Scar coronary bypass scar |
| 48 | True Positive | F | 41.4 | 500 | Underweight | Non verbal controctores upper and lower ext. Cachetic |
| 49 | True Positive | M | 54.5 | 45 | Normal | Frail elderly, NCS |
| 50 | True Positive | F | 50.9 | 243 | Normal | - |
| 51 | True Positive | F | 50.6 | 755 |  | - |
| 52 | True Positive | M | 81.8 | 276 | Overweight | - |
| 53 | True Positive | M | 63.6 | 308 | Normal | Gynecomastia |
| 54 | True Positive | M | 69.1 | 62 | Normal | Mostly mute-moans unsteady ambulation |
| 55 | True Positive | F | 86.4 | 747 | Obese Class I | Requires wheelchair, very frail |
| 56 | True Positive | F | 42.6 | 295 |  | - |
| 57 | True Positive | F | 50 | 318 | Overweight | - |
| 58 | True Positive | F | 52.3 | 266 | Normal | Frail |
| 59 | True Positive | F | 55 | 79 | Normal | Sitting in wheelchair |
| 60 | True Positive | F | 45.5 | 396 | Underweight | Uses walker NCS |
| 61 | True Positive | M | 60 | 60 | Normal | Osteoarthritis, unable to walk. |
| 62 | True Positive | M | 51.8 | 30 | Normal | - |
| 63 | True Positive | F | 85 | 15 | Obese Class II | - |
| 64 | True Positive | M | 59.1 | 42 | Normal | - |
| 65 | True Positive | F | 39.1 | 184 | Underweight | - |
| 66 | True Positive | F | 50 | 193 | Normal | - |
| 67 | True Positive | M | nd | 268 |  | - |
| 68 | True Positive | M | 68.6 | 342 | Normal | - |
| 69 | True Positive | F | 70.5 | 115 | Overweight | Frail elderly |
| 70 | **False Negative** | F | nd | 611 |  | Participant alert, aphasic, will not comply to simple common as |
| 71 | True Positive | F | 78.6 | 189 | Overweight | - |
| 72 | True Positive | M | 72.7 | 397 | Normal | Lying on gurney, mute, immobile |
| 73 | True Positive | F | 72.3 | 155 | Overweight | Lethargic |
| 74 | True Positive | F | 40.9 | 594 | Underweight | - |
| 75 | True Positive | F | 45.9 | 538 | Underweight | Fatal position, NCS |
| 76 | True Positive | F | 63.6 | 180 | Normal | Mildly obtunded |
| 77 | True Positive | F | nd | 200 |  | Patient in fetal position joint contractuores thyroid goiter, right leg amputated below knee. |
| 78 | True Positive | F | 73.6 | 125 | Obese Class I | Sitting in whelchair staring, non-verbal |
| 79 | True Positive | M | 156 | 1 | Obese Class III | Does not open eyes, voluntarily chronic grimace,cachetcic |
| 80 | True Positive | F | 44.5 | 27 | Normal | Contractures, in wheel chair, impaired gait |
| 81 | True Positive | F | 49.1 | 11 | Normal | Somnolent |
| 82 | True Positive | F | 68.2 | 55 | Overweight | - |
| 83 | True Positive | M | 58 | 204 | Underweight | - |
| 84 | True Positive | M | 81.8 | 15 | Overweight | - |
| 85 | True Positive | M | 77.3 | 34 | Normal | Cachectic |
| 86 | True Positive | F | 50.9 | 51 | Normal | - |
| 87 | True Positive | F | 36.4 | 27 | Very severely underweight | Contracted le decubites bilateral heels |
| 88 | True Positive | M | nd | 1 |  | - |
| 89 | True Positive | F | 49.1 | 768 | Underweight | Frail elderly in wheelchair |
| 90 | True Positive | M | 68.2 | 332 | Normal | Generalized weakness/frail, NCS |
| 91 | True Positive | M | nd | 0 |  | Cachectic |
| 92 | True Positive | F | 77.3 | 422 | Obese Class I | - |
| 93 | True Positive | M | 73.2 | 106 | Normal | Decreased nutritional status |
| 94 | True Positive | F | nd | 139 |  | Patient is drows, opens eyesoccassionally to verbal or sensory stimulation |
| 95 | True Positive | M | 77.3 | 181 | Normal | - |
| 96 | True Positive | M | nd | 769 |  | Frail elderly |
| 97 | True Positive | M | 72.7 | 305 | Normal | On stretcher, frail |
| 98 | True Positive | F | 65.9 | 565 | Overweight | Frail |
| 99 | True Positive | M | 72.7 | 846 | Overweight | - |
| 100 | True Positive | F | 60 | 198 | Normal | Sitting in wheelchair does not walk |
| 101 | True Positive | F | 63.6 | 436 | Normal | - |
| 102 | True Positive | F | 57.3 | 66 | Normal | - |
| 103 | True Positive | M | nd | 493 |  | - |
| 104 | True Positive | F | 61.8 | 127 | Normal | - |
| 105 | True Positive | M | 70.9 | 171 | Overweight | - |
| 106 | True Positive | M | 61.8 | 562 | Normal | Sedated |
| Nd – not determined. NCS –NOT CLINICALLY SIGNIFICANT  a PET category by majority PET image assessment and pathology dichotomy normal abnormal by mCERAD_SOT_  b World Health Organisation obesity category based upon BMI  c Assessed at the recruitment sites and not centrally assigned. Therefore may vary between sites | | | | | | |
