# Supplementary material for: Post-mortem histopathology underlying β-amyloid PET imaging following flutemetamol F 18 injection
Source: Acta Neuropathol Commun. 2016 Dec 12;4:130. doi: 10.1186/s40478-016-0399-z (PMC5154022; doi:10.1186/s40478-016-0399-z)
Supplement: Additional file 6: — Amyloid plaque frequencies. In addition to the neuritic plaque frequencies assessed on Bielschowsky silver stained sections, semiquantitative plaque frequencies of none, sparse, moderate or frequent [43] were obtained from β-amyloid stained sections. In addition to the frequencies recorded in the CERAD neocortical regions, the Amyloid phase was recorded after examining multiple cortical, subcortical, midbrain and hindbrain regions [58]. (DOC 112 kb) [file 40478_2016_399_MOESM6_ESM.doc]

| **Clinical Status of subjects at PET Scan** | | | | |
| --- | --- | --- | --- | --- |
| **Case** | **Amyloid Phase** | **Frontal lobe** | **Parietal Lobe** | **Temporal lobe** |
| 1 | 1 | Sparse | Sparse | None |
| 2 | 0 | None | None | None |
| 3 | 1 | None | None | Sparse |
| 4 | 0 | None | None | None |
| 5 | 1 | Sparse | None | Sparse |
| 6 | 1 | Sparse | None | Sparse |
| 7 | 0 | None | None | None |
| 8 | 0 | None | None | None |
| 9 | 0 | None | None | None |
| 10 | 1 | Sparse | Sparse | None |
| 11 | 1 | None | Sparse | None |
| 12 | 1 | Sparse | None | Sparse |
| 13 | 2 | Frequent | Sparse | Moderate |
| 14 | 0 | None | None | None |
| 15 | 2 | Sparse | Sparse | Sparse |
| 16 | 0 | None | None | None |
| 17 | 1 | Sparse | None | None |
| 18 | 1 | Sparse | None | Moderate |
| 19 | 2 | None | Sparse | Sparse |
| 20 | 1 | Sparse | None | Sparse |
| 21 | 4 | Frequent | Frequent | Frequent |
| 22 | 2 | Sparse | Sparse | Moderate |
| 23 | 3 | Sparse | Moderate | Frequent |
| 24 | 3 | Moderate | Moderate | Frequent |
| 25 | 2 | Frequent | Frequent | Frequent |
| 26 | 4 | Moderate | Sparse | Moderate |
| 27 | 4 | Moderate | Moderate | Moderate |
| 28 | 3 | Moderate | Moderate | Moderate |
| 29 | 5 | Frequent | Frequent | Frequent |
| 30 | 5 | Frequent | Frequent | Frequent |
| 31 | 4 | Frequent | Frequent | Frequent |
| 32 | 5 | Frequent | Frequent | Frequent |
| 33 | 4 | Frequent | Frequent | Frequent |
| 34 | 5 | Frequent | Frequent | Frequent |
| 35 | 5 | Frequent | Frequent | Frequent |
| 36 | 5 | Frequent | Frequent | Frequent |
| 37 | 5 | Frequent | Frequent | Frequent |
| 38 | 3 | Moderate | Moderate | Moderate |
| 39 | 3 | Moderate | Moderate | Moderate |
| 40 | 3 | Moderate | Sparse | Moderate |
| 41 | 3 | Frequent | Moderate | Moderate |
| 42 | 4 | Frequent | Frequent | Frequent |
| 43 | 4 | Frequent | Frequent | Frequent |
| 44 | 3 | Frequent | Frequent | Frequent |
| 45 | 5 | Frequent | Frequent | Frequent |
| 46 | 4 | Frequent | Frequent | Frequent |
| 47 | 5 | Frequent | Frequent | Frequent |
| 48 | 3 | Moderate | Moderate | Moderate |
| 49 | 3 | Moderate | Moderate | Moderate |
| 50 | 5 | Frequent | Frequent | Frequent |
| 51 | 5 | Frequent | Frequent | Frequent |
| 52 | 4 | Frequent | Frequent | Frequent |
| 53 | 4 | Frequent | Frequent | Frequent |
| 54 | 5 | Frequent | Frequent | Frequent |
| 55 | 3 | Moderate | Moderate | Moderate |
| 56 | 3 | Moderate | Frequent | Frequent |
| 57 | 5 | Frequent | Frequent | Frequent |
| 58 | 4 | Frequent | Moderate | Moderate |
| 59 | 5 | Frequent | Frequent | Frequent |
| 60 | 5 | Moderate | Frequent | Moderate |
| 61 | 5 | Frequent | Frequent | Frequent |
| 62 | 4 | Moderate | Moderate | Frequent |
| 63 | 5 | Frequent | Frequent | Frequent |
| 64 | 4 | Frequent | Frequent | Frequent |
| 65 | 3 | Frequent | Frequent | Frequent |
| 66 | 5 | Frequent | Frequent | Frequent |
| 67 | 5 | Frequent | Frequent | Frequent |
| 68 | 5 | Frequent | Frequent | Frequent |
| 69 | 5 | Frequent | Frequent | Frequent |
| 70 | 4 | Frequent | Frequent | Frequent |
| 71 | 3 | Frequent | Frequent | Frequent |
| 72 | 5 | Frequent | Frequent | Frequent |
| 73 | 5 | Frequent | Frequent | Frequent |
| 74 | 5 | Frequent | Frequent | Frequent |
| 75 | 4 | Frequent | Frequent | Frequent |
| 76 | 4 | Frequent | Frequent | Frequent |
| 77 | 5 | Frequent | Frequent | Frequent |
| 78 | 5 | Frequent | Frequent | Frequent |
| 79 | 5 | Frequent | Frequent | Frequent |
| 80 | 5 | Frequent | Frequent | Frequent |
| 81 | 4 | Frequent | Frequent | Frequent |
| 82 | 4 | Frequent | Frequent | Frequent |
| 83 | 4 | Frequent | Frequent | Frequent |
| 84 | 4 | Frequent | Frequent | Frequent |
| 85 | 5 | Frequent | Frequent | Frequent |
| 86 | 4 | Frequent | Frequent | Frequent |
| 87 | 5 | Frequent | Frequent | Frequent |
| 88 | 4 | Frequent | Frequent | Moderate |
| 89 | 5 | Frequent | Frequent | Frequent |
| 90 | 5 | Moderate | Frequent | Moderate |
| 91 | 5 | Frequent | Frequent | Frequent |
| 92 | 5 | Frequent | Frequent | Frequent |
| 93 | 5 | Frequent | Frequent | Frequent |
| 94 | 5 | Frequent | Frequent | Frequent |
| 95 | 4 | Frequent | Frequent | Frequent |
| 96 | 5 | Frequent | Frequent | Frequent |
| 97 | 5 | Frequent | Frequent | Frequent |
| 98 | 5 | Frequent | Frequent | Frequent |
| 99 | 5 | Frequent | Frequent | Frequent |
| 100 | 4 | Frequent | Frequent | Frequent |
| 101 | 5 | Frequent | Frequent | Frequent |
| 102 | 5 | Frequent | Frequent | Frequent |
| 103 | 5 | Frequent | Frequent | Frequent |
| 104 | 5 | Frequent | Frequent | Frequent |
| 105 | 5 | Frequent | Frequent | Frequent |
| 106 | 5 | Frequent | Frequent | Frequent |
